# De‐Saturation of Single‐Atom Copper Catalysts for Accelerating Propargylic Substitution Reactions

**DOI:** 10.1002/adma.202509221

**Published:** 2025-08-25

**Authors:** Qilong Cai, Yang Meng, Chao Wu, Wenjia Qu, Qiang Wang, Tan Li, Chengyi Liu, Jinxing Chen, Huihui Lin, Qian He, Yafei Zhao, Shibo Xi, Jiong Lu

**Affiliations:** ^1^ Department of Chemistry National University of Singapore Singapore 117543 Singapore; ^2^ National University of Singapore (Suzhou) Research Institute No. 377 Linquan Street Suzhou Jiangsu P. R. China; ^3^ Institute of Sustainability for Chemicals, Energy and Environment (ISCE2) Agency for Science, Technology and Research (A*STAR) 1 Pesek Road Jurong Island Singapore 627833 Singapore; ^4^ College of Materials Science and Engineering Sichuan University Chengdu 610065 P. R. China; ^5^ Department of Materials Science and Engineering National University of Singapore Singapore 117574 Singapore; ^6^ State Key Laboratory of Precision and Intelligent Chemistry University of Science and Technology of China (USTC) Jinzhai Road 96 Hefei Anhui 230026 P. R. China

**Keywords:** de‐saturation strategy, KOH‐mediated Joule thermal, propargylic substitution reactions, single‐atom catalysts

## Abstract

Rational design of proximal coordination microenvironments surrounding catalytic sites to achieve optimal reaction kinetics represents a paramount pursuit in single‐atom catalysts (SACs), yet continues to pose substantial synthetic challenges. Developing innovative strategies that simultaneously stabilize low‐coordinated single‐metal species on solid supports, while ensuring atomic precision and high activity, remains imperative. Herein, a de‐saturation strategy for SACs is demonstrated (denoted as De‐sat SACs) using a top‐down approach based on a KOH‐mediated Joule thermal shock to obtain under‐coordinated and asymmetric SACs for efficient organic synthesis. Using copper‐based SACs as a proof‐of‐concept, the de‐saturation strategy effectively converts the CuN_4_ to CuN_3_ configuration. The De‐sat Cu SACs exhibit remarkable catalytic activity in propargylic substitution reactions, tolerating a broad range of nucleophiles (N–, C–, and O–), as well as diverse aryl, alkyl, tertiary, and cyclic propargylic carbonates. The coordination reduction in these De‐sat SACs not only breaks the structural symmetry to enhance site accessibility but also elevates the energy of the $d_{z^{2}}$ orbital of Cu atom, thereby facilitating the formation of copper–alkynyl intermediates and boosting their catalytic performance. These findings establish a new platform for the rational design and synthesis of de‐saturated yet stable SACs, facilitating challenging catalytic transformations toward sustainable chemical manufacturing.

## Graphical abstract

De‐saturation of copper‐based single‐atom catalysts (De‐sat SACs) are realized by a KOH‐mediated Joule thermal shock strategy. Benefiting from the under‐saturation coordination environment of single‐atom Cu center, De‐sat Cu SACs exhibit remarkable catalytic activity in propargylic substitution reactions, tolerating a broad range of nucleophiles (N–, C–, and O–), as well as diverse aryl, alkyl, tertiary, and cyclic propargylic carbonates.

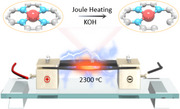

## 1 Introduction

Single‐atom catalysts (SACs) have garnered extensive attention due to their maximized atom utilization efficiency and well‐defined active sites, offering an ideal platform for mechanistic understanding and catalytic innovation for sustainable organic synthesis.^[^ ^1^, ^2^, ^3^, ^4^, ^5^, ^6^ ^]^ Generally, the support design must maintain the stability of the metal center while also providing enough structural flexibility to enable an efficient catalytic cycle. However, the chemical bonds between the metal centers and the support, which are essential for preventing metal aggregation, can introduce spatial constraints (coordination near‐saturation) that hinder the activation and adsorption of reactants in multi‐step organic reactions.^[^ ^7^, ^8^ ^]^ Taking M‐N_4_ as one representative case, single metal atoms in these SACs are stabilized by nearly‐saturated, symmetric coordination environments, which not only provide high thermodynamic stability but also limit the electronic tunability and catalytic activity of such active centers.^[^ ^9^, ^10^, ^11^, ^12^ ^]^ Therefore, the structure of these nearly or fully saturated mononuclear metal species, stabilized on solid supports, is typically suboptimal for catalyzing complex organic transformations.

It is widely anticipated that the degree of saturation and local bonding symmetry of SACs are crucial in determining their stability and catalytic performances,^[^ ^13^, ^14^, ^15^, ^16^, ^17^, ^18^, ^19^, ^20^, ^21^, ^22^ ^]^ as also predicted in recent studies.^[^ ^23^, ^24^, ^25^ ^]^ Reducing the coordination number along with modified asymmetry in SACs can unlock unique electronic structures and catalytic behaviors that are not achievable with symmetric, highly coordinated single‐atom motifs. However, the rational and controllable fabrication of low‐saturation, asymmetric single‐atom sites remains a significant synthetic challenge. To date, most efforts to generate low‐coordinated or asymmetric SACs have relied on bottom‐up approaches, including defect engineering, support/ligand design, or precursor modulation, which often suffer from limited structural precision and poor scalability.^[^ ^26^, ^27^, ^28^ ^]^ Moreover, the uncontrolled generation of defects or the risk of aggregation during these processes can obscure the intrinsic structure‐activity relationships.^[^ ^29^, ^30^, ^31^ ^]^

To address these challenges, we introduced a de‐saturation concept for SACs (De‐sat SACs) using a top‐down strategy, which enables the reduction of coordination number and the realization of desired asymmetry in pre‐synthesized SAC motifs in a controllable and scalable manner. Specifically, we have transformed the pre‐synthesized Cu SACs with well‐established CuN_4_ sites into asymmetric De‐sat Cu SACs with engineered CuN_3_ sites through this rapid top‐down de‐saturation approach. By intimately mixing Cu SACs with potassium hydroxide (KOH) and subjecting the mixture to rapid Joule heating, the coordination environment around Cu centers can be precisely modulated, selectively removing one nitrogen without disrupting atomic dispersion. The chemical interaction between KOH and the Cu SACs framework enables targeted deconstruction at high‐temperature within milliseconds, resulting in the formation of robust de‐saturated Cu single‐atom catalysts (De‐sat Cu SACs). Based on this rational design, the De‐sat Cu SACs have proven to be highly efficient heterogeneous catalysts for propargylic substitution reactions. Such a strategy holds great promise for creating de‐saturated yet robust SACs, capable of tackling challenging organic transformations.

## 2 Results and Discussion

**Figure 1:**
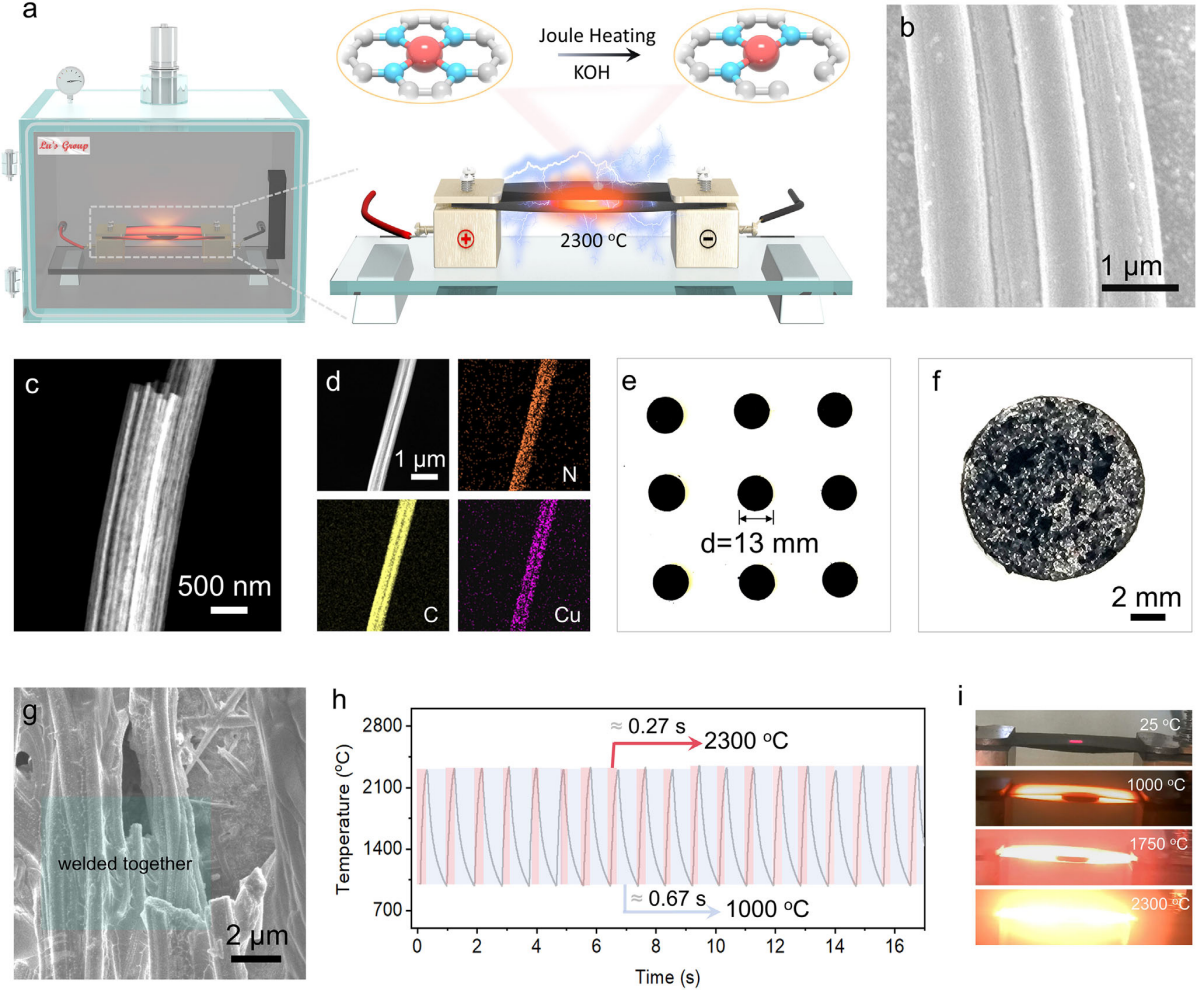
a) Schematic illustration of the preparation strategy for the original Cu SACs and De‐sat Cu SACs through rapid Joule Heating. b) FESEM and c) Dark‐field TEM images of Cu SACs. d) HAADF‐STEM and the corresponding elemental mapping images of Cu SACs. e,f) Photograph and g) FESEM images of the pressed black pellet of the mixture KOH and Cu SACs powders. h) Cyclic shock heating patterns. i) Photograph of the desaturation of Cu SACs at different temperatures under Joule heating.

The preparation of De‐sat Cu SACs was carried out in a Joule‐heated^[^ ^32^ ^]^ reactor (**Figure** **1**a; Figure S1a,b, Supporting Information). Prior to this, we fabricated the catalyst support using polyacrylonitrile (PAN) and polystyrene (PS) fibers as the precursors. This choice was based on the fibers' high nitrogen content and large specific surface area, making them ideal candidates to fabricate the support for anchoring copper atoms. The PAN/PS fibers synthesized here exhibit highly uniform solid structures, with an average diameter of ≈2 µm, as revealed in both field‐emission scanning electron microscopy (FESEM) (Figure S2a,b, Supporting Information) and transmission electron microscopy (TEM) imaging (Figure S2c, Supporting Information).

After carbonization at 1000 ^°^C under an argon atmosphere, the PAN/PS fibers were converted into nitrogen‐doped amorphous carbon multichannel fibers (CMFs) (Figure S3a,b, Supporting Information), which will be used as the support to anchor Cu single atoms. The FESEM image reveals that the CMFs possess a uniform, smooth fibrous morphology with submicron‐scale diameters (Figure S3c, Supporting Information). The X‐ray diffraction (XRD) pattern of CMFs exhibits a more pronounced peak at ≈25.3°, compared to the PAN/PS fibers (Figure S3d, Supporting Information), indicating that the carbonization treatment produces partially amorphous CMFs with graphite‐like microcrystalline structures.^[^ ^33^, ^34^, ^35^ ^]^ The Raman spectra of CMFs exhibit the D band (≈1350 cm^−1^), associated with structural disorder or defects, and the G band (≈1580 cm^−1^), corresponding to in‐plane phonon mode of sp^2^‐bonded carbon. Together, these features reflect the crystallinity and defect density of such carbon supports (Figure S3e, Supporting Information). EDX analysis confirms that carbon constitutes the primary component of the CMFs, with successful incorporation of nitrogen. The carbon and nitrogen contents are ≈98.28% and 1.72%, respectively (Figure S3f,g,h, Supporting Information).

Subsequently, Cu SACs were fabricated through a typical copper ion impregnation process, followed by high‐temperature annealing. As‐prepared Cu SACs retain the fiber‐like morphology (Figure 1b) with the multichannel structure (Figure 1c). The atomic dispersion of Cu SACs has been verified by various characterization techniques including high angle annular dark field scanning transmission electron microscopy (HAADF‐STEM) and X‐ray absorption spectroscopy (XAS) (Figure 1c). HAADF‐STEM and corresponding elemental mapping images revealed the uniform distribution of C, N, and Cu elements within individual Cu SACs (Figure 1d), in contrast to randomly distributed Cu nanoparticles observed in Cu NPs@CMFs (Figure S4, Supporting Information). In addition, the Cu content in Cu SACs was determined to be ≈1.17 wt.% (Table S1, Supporting Information) using the inductively coupled plasma optical emission spectroscopy (ICP‐OES).

To prepare the De‐sat Cu SACs, KOH was employed as the etching reagent. To enhance contact between the etchant and the as‐obtained Cu SACs, large KOH flakes were first ground into a fine powder and then thoroughly mixed with the Cu SACs‐loaded multichannel fibers. This mixture was subsequently compressed into black tablets ≈13 mm in diameter (Figure 1e). Upon closer examination, many fine KOH particles have successfully enveloped and fused with the Cu SACs‐loaded multichannel fibers (Figure 1f,g). To conduct the Joule‐heating, a pressed black pellet composed of mixed KOH and Cu SACs powders was sandwiched between two Joule‐heating carbon strips. This enables a swift heating to create a uniform high‐temperature environment for fast etching of Cu SACs (Figure 1h). As the temperature and time increase, the carbon paper gradually turns from black to red‐hot (Figure 1i). A high‐temperature of up to ≈2300 °C can be achieved within 0.27 s, which was sufficient to provide the energy required to initiate chemical reactions between KOH and Cu SACs materials or the Cu SACs‐loaded multichannel fibers.

**Figure 2:**
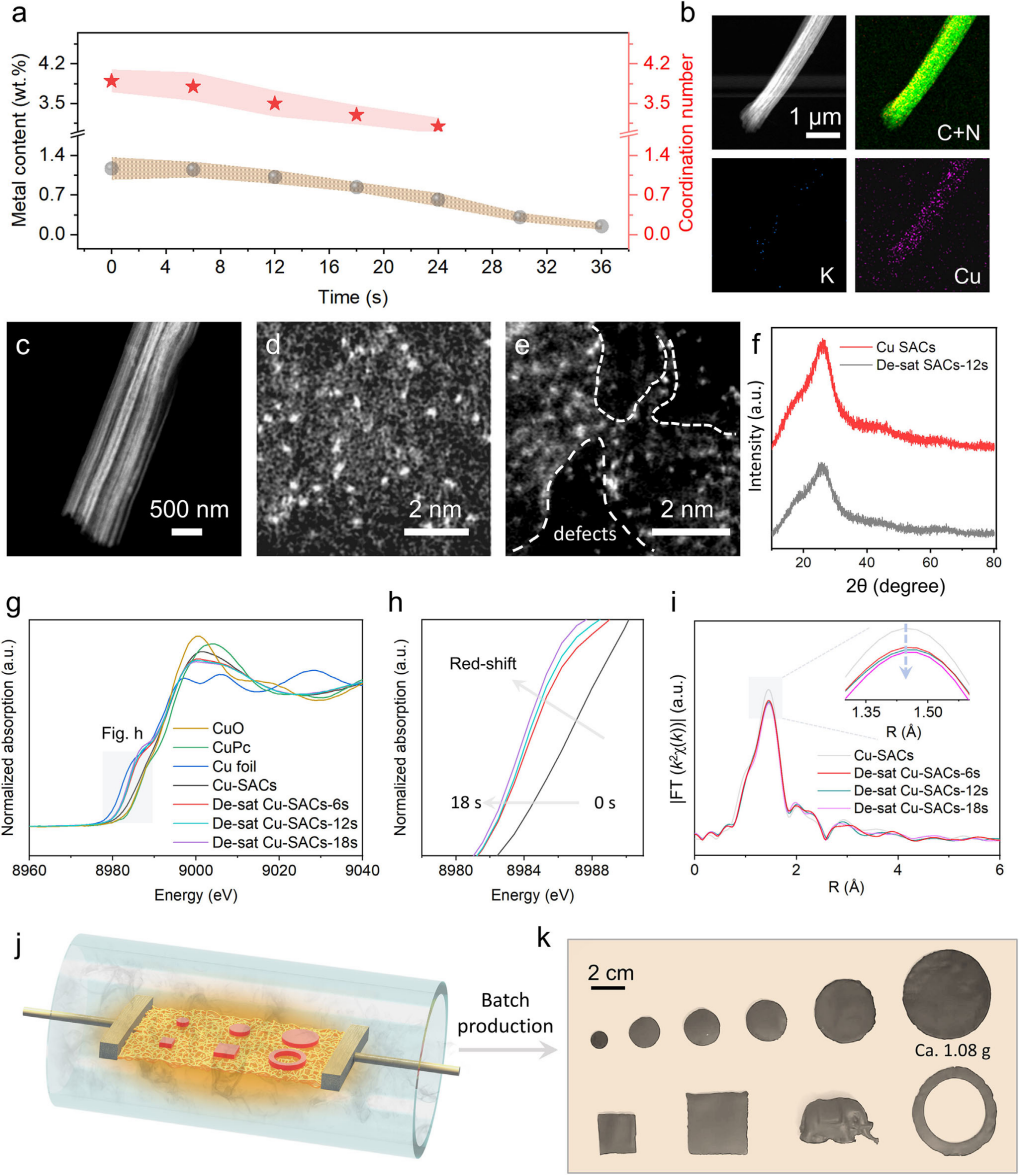
a) Plots of metal content and coordination number as a function of reaction time. b) HAADF‐STEM and the corresponding elemental mapping images of a single De‐sat Cu SACs. c) HAADF‐STEM image of De‐sat Cu SACs‐12s. Aberration‐corrected HAADF‐STEM images of d) Cu SACs and e) De‐sat Cu SACs‐12s. f) XRD pattern of De‐sat Cu SACs‐12s. g–i) X‐ray absorption spectra of Cu SACs and De‐sat Cu SACs with different etching times. j,k) Schematic of a batch production of tablets of different sizes and shapes.

We then investigated the potential relationship between metal loading/coordination number of Cu‐based SACs as a function of etching duration. It is found that as the etching time increased, the content of Cu gradually decreased (**Figure** **2**a). Specifically, it dropped from 1.17 wt.% in the original Cu SACs to 1.15 wt.% after 6 s of etching, 1.02 wt.% after 12 s, and further to 0.84 wt.% after 18 s (Table S1, Supporting Information). After 24 s of etching, the Cu content had decreased by ≈87%. The rapid release of Joule heat, combined with the interaction between KOH and the support, is likely to alter the coordination environment of Cu species.

We note that the De‐sat Cu SACs retain their distinct multichannel structure and exhibit uniform distribution of C, N, and Cu elements (Figure 2b,c; Figure S5, Supporting Information), with no evidence of aggregated Cu nanoparticles (Figure 2f). Additionally, the absence of potassium‐related signals in the sample etched for 12 s (denoted as De‐sat Cu SACs‐12s) indicates that potassium was not incorporated into the support. HAADF–STEM imaging reveals that both Cu SACs and de‐saturated Cu SACs display numerous isolated bright dots (Figure 2d,e), indicating that the atomic dispersion of Cu single atoms is preserved before and after etching. Instead, the rapid heating primarily induced alterations in the micro‐scale morphology and overall porosity within the support material, as revealed in the N_2_ adsorption‐desorption isotherms and BET surface area measurements. Specifically, De‐sat Cu SACs‐12s (31.6 m^2^ g^−1^) exhibit a significantly higher surface area compared to Cu SACs (26.1 m^2^ g^−1^) (Figure S6, Supporting Information).

To gain deeper insights into the atomic coordination of Cu single atoms, we conducted a detailed analysis of XAS spectra of both Cu SACs and several De‐sat Cu SACs samples. As depicted in Figure 2g, the absorption thresholds of Cu SACs and De‐sat Cu SACs (dark gray region) lie between those of copper foil and copper oxide (CuO), indicating the presence of copper in positive valence states. Intriguingly, increasing the etching time induces a pronounced redshift in the absorption threshold, suggesting that the etching process transforms the valence state of Cu from +2 to +1 (Figure 2h). Additionally, a slight reduction in the peak intensity directly correlates with a marked decrease in the number of first‐shell coordination atoms. Specifically, the Fourier transform spectra of the Extended X‐ray Absorption Fine Structure (EXAFS), reveal one dominant peak ≈1.5 Å, which can be assigned to the Cu–N bond (Figure 2i).

Furthermore, the intensity of the corresponding peaks gradually decreases as the etching time increases, thus indicating a decrease in the Cu–N coordination number. The corresponding fitting results (Figure S7a–f; Table S2, Supporting Information) reveal that the Cu─N coordination number is reduced from ≈4 in Cu SACs to ≈3 in De‐sat Cu SACs‐12s. All the characterization results point out that KOH‐mediated Joule thermal shock treatment modifies the Cu SACs with CuN_4_ sites into De‐sat Cu SACs with CuN_3_ sites. In addition, we fabricated and tested large‐scale samples to verify the scalability of this desaturation approach—an essential step toward future large‐scale applications. Notably, the largest sample prepared, measuring ≈5 cm in diameter and 0.3 cm in thickness, contained 1.08 g of catalyst (Figure 2j). Furthermore, ultrafast high‐temperature heating lasting only a few seconds effectively suppresses byproduct formation and enables the rapid synthesis of catalysts with desired local atomic structure and global geometries (Figure 2k).

De‐sat Cu SACs with a reduced degree of saturation are expected to hold great potential in organic synthesis. Despite considerable achievements in copper‐catalyzed propargylic substitution reactions employing homogeneous catalysts,^[^ ^36^, ^37^, ^38^, ^39^, ^40^, ^41^, ^42^, ^43^ ^]^ persistent challenges remain, including their high production costs, environmental burdens arising from inefficient separation, purification processes and difficulties in catalyst recycling.^[^ ^44^, ^45^ ^]^ Although propargylic compounds serve as key motifs in natural products, pharmaceuticals and valuable synthetic intermediates,^[^ ^46^ ^]^ the development of heterogeneous catalysts for their construction remains scarce and relatively underexplored.^[^ ^47^ ^]^ A central challenge lies in the formation of reactive copper–alkynyl and copper–allenylidene intermediates, which are crucial for activating propargyl carbonates and facilitating C–C and C–heteroatom bond formation.^[^ ^42^ ^]^ In typical heterogeneous systems—such as those based on CuN_4_ sites in defective graphene nanosheets,^[^ ^48^ ^]^ carbon nitrides,^[^ ^49^ ^]^ or nitrogen‐doped carbons,^[^ ^50^ ^]^ the highly saturated and rigid coordination environments often hinder the formation of low‐valent, low‐coordinate copper species required for propargyl carbonates activation. Reducing the coordination number of heterogeneous copper catalysts may offer an effective strategy to enable such transformations.

**Figure 3:**
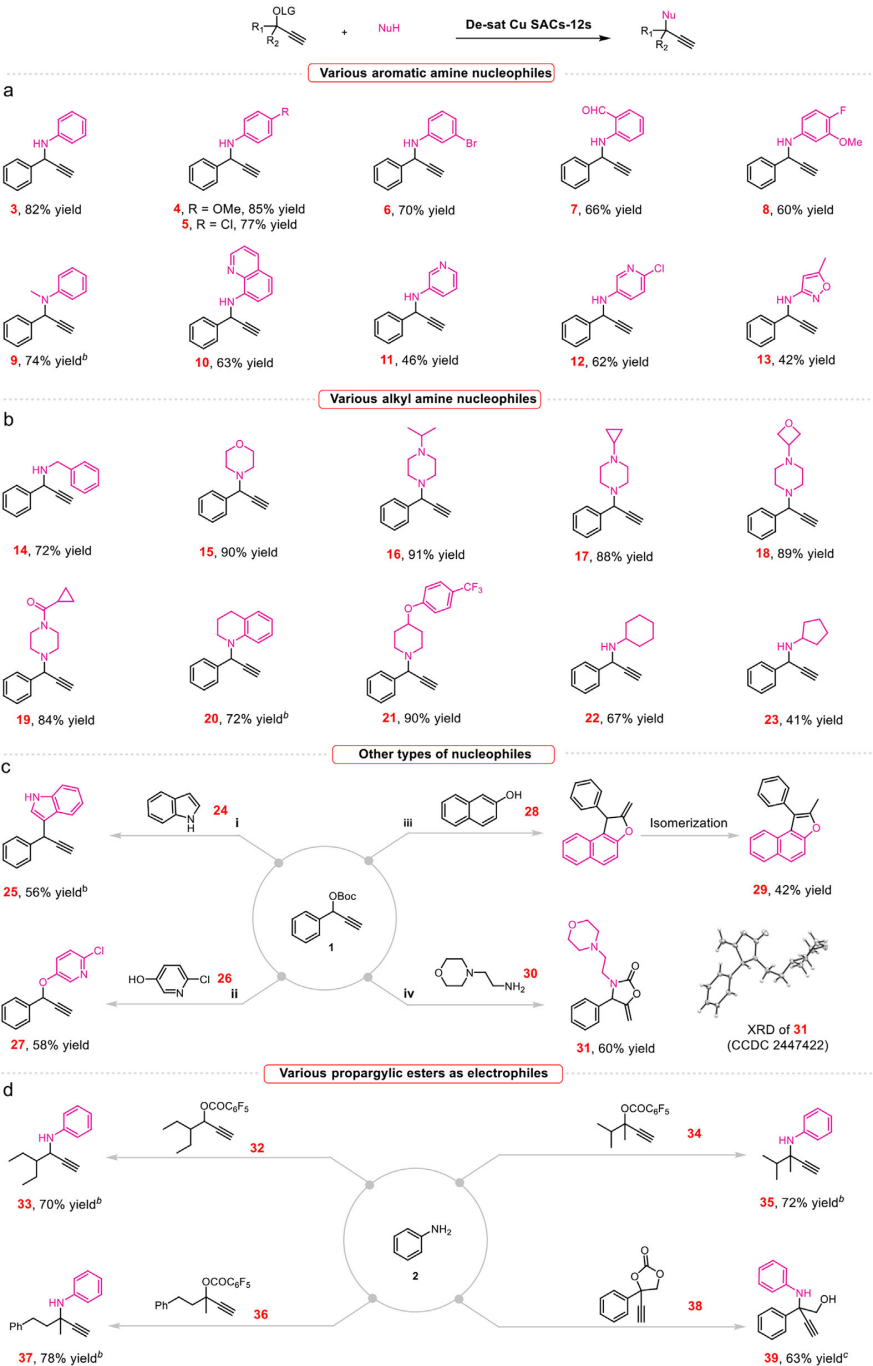
The evaluation of substrate scopes of De‐sat Cu SACs‐12s. a) Various aromatic amine nucleophiles. b) Various alkyl amine nucleophiles. c) Other types of nucleophiles. d) Various propargylic esters as electrophiles. Reaction conditions: propargyl carbonates (0.1 mmol), nucleophiles (0.2 mmol), Et_3_N (2.0 equiv), De‐sat Cu SACs‐12s (1.0 mmol%), MeOH (0.5 mL), 60 °C, 24 h, isolated yield. *^b^* De‐sat Cu SACs‐12s (2.0 mmol%). *^c t^* BuOLi (2.0 equiv) instead of Et_3_N (2.0 equiv) as base.

Next, we investigated the catalytic performance of De‐sat Cu SACs‐12s in the catalytic propargyl substitution reaction of propargyl carbonates (**1**) with various nucleophiles (**2**). The reaction proceeded smoothly in MeOH at 60 °C with 1.0 mol% De‐sat Cu SACs‐12s as the catalyst and triethylamine (Et_3_N) as the base. The reaction produced the desired product **3** in 82% yield after 24 h, demonstrating the feasibility of using de‐saturated Cu SACs‐12s for this reaction (**Figure** **3**a). Additionally, De‐sat Cu SACs‐12s also exhibits a broad substrate scope of various aromatic amine nucleophile under the optimized reaction conditions. For example, the substrates **2** bearing electron‐donating (–OMe) or electron‐withdrawing substituents (–Cl) at the *para*‐position of the aryl group proceed well, affording the corresponding products **4**–**5** in 77–85% yields. Substrates bearing *meta*–Br and *ortho*–CHO substituted aryl amines also reacted well, producing **6**–**7** in good yields (66%–70%). The effectiveness of this system was further demonstrated with the reactions of poly‐substituted aryl amine, leading to the corresponding product **8** in 60% yield. Besides the aniline nucleophiles, *N*–methylaniline was also compatible with the reaction conditions, affording the desired product **9** in 74% yield. Notably, more challenging heteroaryl amines were also explored, providing the corresponding products **10**–**13** in moderate yields (42%–63%). In addition to aryl and heteroaryl amines, various classes of alkyl amines were also carefully investigated to assess the generality of the method (Figure 3b). Notably, benzylamine (**14**), morpholine (**15**), piperazine (**16**–**19**), piperidine (**20**–**21**), cyclohexylamine (**22**) and cyclopentylamine (**23**) were also amenable to the reaction, giving the corresponding products **14**–**23** in 41%–91% yields.

We also found that the reaction was not restricted to amines; for instance, the C–3 position of indole (**24**) acted as an effective C–nucleophile, affording the corresponding product **25** in 56% yield (Figure 3c–i). 6‐chloropyridine‐3‐ol (**26**) was also a compatible O–nucleophile, leading to the desired product **27** in 58% yield (Figure 3c–ii). Notably, the methodology was applicable to the reaction of **1** with 1,3‐C,O‐bis‐nucleophiles such as 2‐naphthol (**28**), leading to the formation of the target [3+2]–annulation product, followed by terminal alkene isomerization to afford product **29** in 42% yield (Figure 3c–iii). Surprisingly, when 4‐(2‐aminoethyl)morpholine (**30**) was employed as the N–nucleophile, the tandem propargylic amination–carboxylative cyclization sequence toward product **31** featuring an exocyclic methylene moiety was obtained in 60% yield via a one‐pot reaction (Figure 3c–iv). The structure of **31** was further identified by X‐ray crystal structural analysis (Figure S8 and Table S3, Supporting Information).

Besides the aryl propargylic carbonate **1**, more challenging propargylic substrate **32** bearing alkyl groups at the propargylic position was also carefully investigated (Figure 3d). The substrate **32** with –OCOC_6_F_5_ group gave the desired propargylamine **33** (70% yield). Encouraged by the efficiency of the De‐sat Cu SACs‐12s catalysts, we further evaluated its performance in the more challenging substitution reactions involving tertiary propargylic esters, where increased steric hindrance complicates the reaction process. Remarkably, both substrates underwent the transformation efficiently, delivering the desired products **35** and **37** in 72%–78% yields. Intriguingly, the reaction of propargylic cyclic carbonate **38** with aniline leads to the formation of the of *β*–Amino alcohol **39** in 63% yield. In addition, we found that the reaction failed to proceed with internal propargylic esters under the same conditions. Furthermore, no product formation was observed when the substrate contained a thiol (–SH) group, suggesting that strongly coordinating functional groups can significantly inhibit the reaction, likely by binding to the metal center and disrupting the catalytic cycle (Figure S9, Supporting Information).

**Figure 4:**
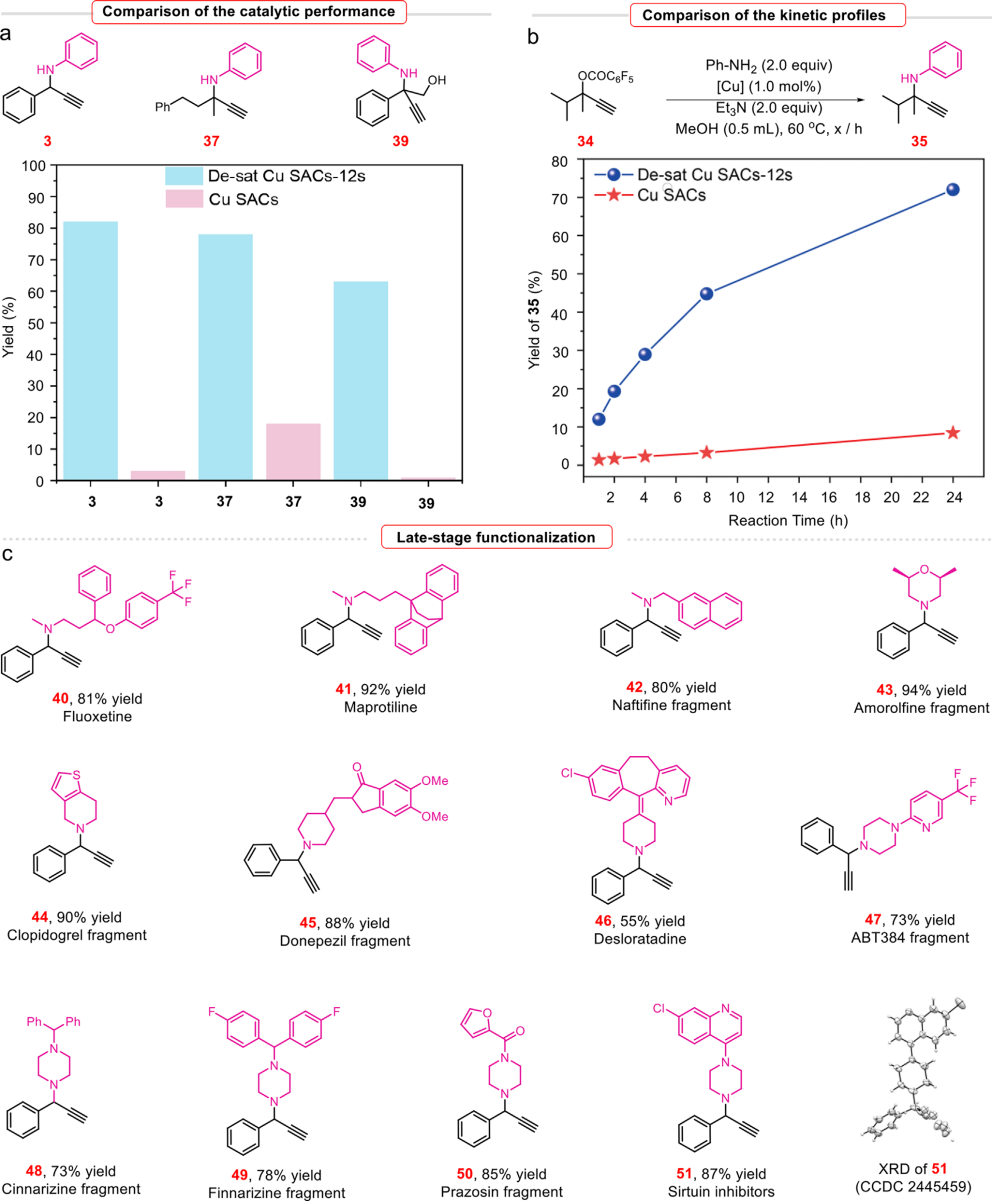
High‐performance of De‐sat Cu SACs‐12s. a) Comparison of the catalytic performance. b) Comparison of the kinetic profiles. c) Late‐stage functionalization. Reaction conditions: propargyl carbonates (0.1 mmol), nucleophiles (0.2 mmol), Et_3_N (0.2 mmol), De‐sat Cu SACs‐12s (1.0 mmol%), MeOH (0.5 mL), 60 ^°^C, 24 h, isolated yield.

The high‐performance of De‐sat Cu SACs‐12s were further demonstrated by evaluating the catalyst in various synthetic scenarios. For the three different propargylic carbonates, we first evaluated the catalytic performance of Cu SACs and De‐sat Cu SACs‐12s catalysts under standard conditions (**Figure** **4**a; Table S4, Supporting Information). The original Cu SACs exhibited limited catalytic activity. In contrast, De‐sat Cu SACs‐12s significantly enhanced catalytic efficiency in the propargylic substitution reaction. The use of cyclohexylamine as a nucleophile further demonstrated the superior catalytic efficiency of De‐sat Cu SACs compared to Cu SACs and other representative heterogeneous copper catalysts (Table S5, Supporting Information). Subsequently, several batches of the same reaction were carried out in parallel under identical conditions using De‐sat Cu SACs‐12s and Cu SACs, respectively (Figure 4b; Table S6, Supporting Information). The reactions were quenched at different time intervals in order to monitor and compare the reaction kinetics. The reaction kinetics studies once again demonstrate that the De‐sat Cu SACs‐12s catalyst possesses superior catalytic activity relative to Cu SACs, thereby highlighting that the etching process leads to a significant enhancement in catalytic performance. In addition, De‐sat Cu SACs‐12s demonstrated excellent durability and recyclability in the propargyl substitution reaction as shown with no appreciable conversion and selectivity decrease over five consecutive runs (Figure S10, Supporting Information). We also obtained TEM, HAADF‐STEM, and selected‐area electron diffraction (SAED) images, as well as XRD pattern of De‐sat Cu SACs‐12s after reaction, revealing the retained structural integrity, atomic dispersion of Cu species, and crystalline characteristics of the post‐reaction catalyst (for the details, refer to Figure S11, Supporting Information).

The synthetic potential of this transformation was further demonstrated through late‐stage functionalization of selected pharmaceuticals and their derivatives (Figure 4c). Notably, both acyclic (**40**–**42**) and cyclic bioactive amines (**43–51**) underwent smooth transformations, thereby highlighting the broad applicability and utility of this protocol for the construction of structurally complex propargylamine derivatives. Fluoxetine, a widely prescribed selective serotonin reuptake inhibitor (SSRI), was employed as the alkyl amine in the reaction with compound **1**, affording the corresponding product **40** in 81% yield. Similarly, maprotiline, a tetracyclic antidepressant (TeCA), underwent a smooth transformation, furnishing product **41** in an excellent yield (92%).

Other acyclic bioactive amines, such as naftifine and amorolfine fragment, were also competent coupling partners, providing the corresponding product **42**–**43** in 80%–94% yields. A diverse set of piperidine‐based substrates, such as clopidogrel, donepezil fragments and desloratadine, reacted efficiently with **1** to furnish the corresponding products **44**–**46** in moderate to high yields (55–90%). Given the medicinal significance of piperidine and piperazine cores, we tested a broad range of drugs (or their fragments) containing these motifs in this amination reaction, which led to the corresponding complex propargylic amines **47**–**51** in good yields (73%–87%). The structure of product **51** was further confirmed by X‐ray crystallographic analysis (Figure S12 and Table S7, Supporting Information). These results highlight the outstanding catalytic performance of the De‐sat Cu SACs‐12s catalysts in the current reaction.

**Figure 5:**
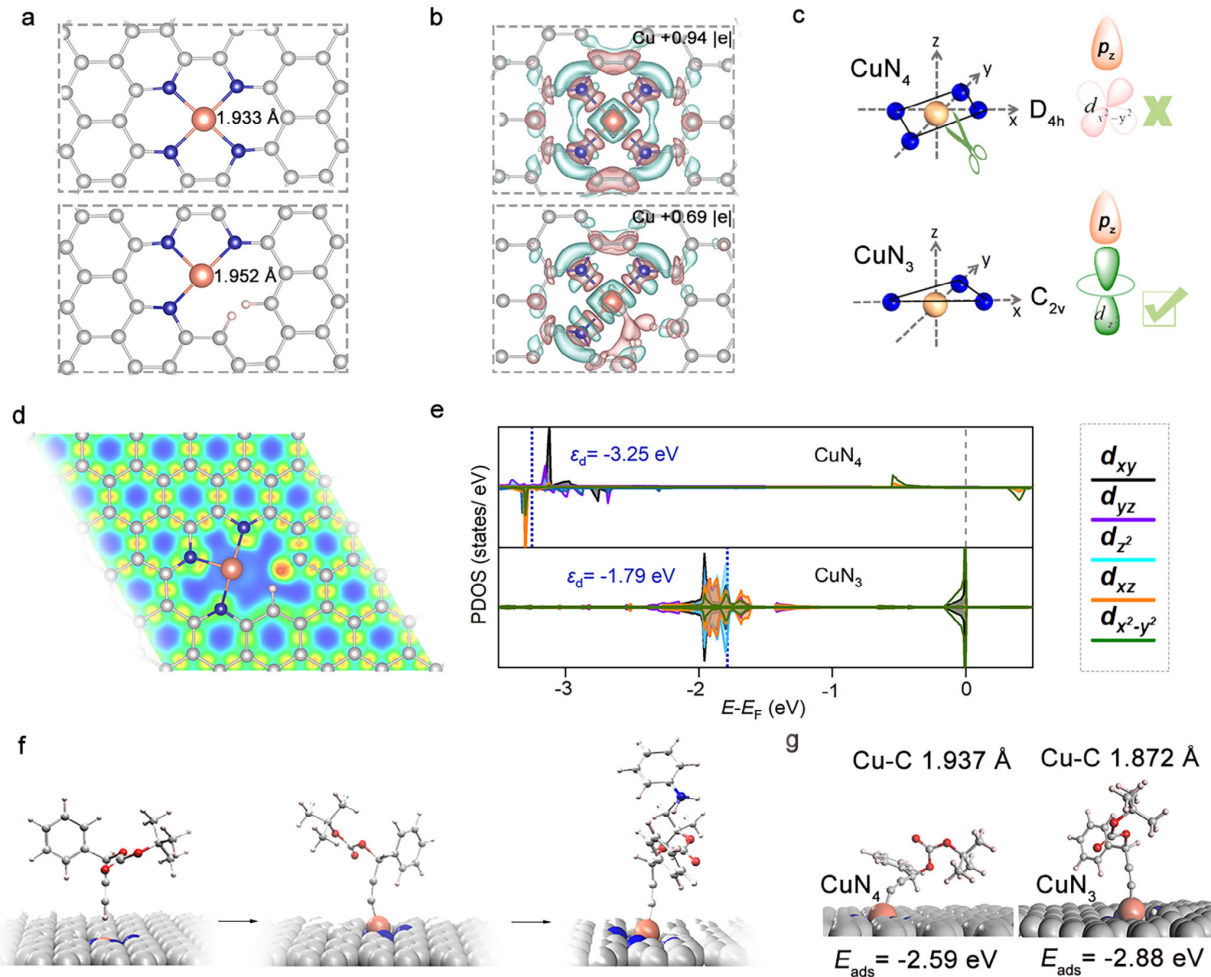
a) Theoretical models of Cu SACs (CuN_4_) and De‐sat Cu SACs (CuN_3_) active sites. b) Charge density difference plots of Cu SACs and De‐sat Cu SACs (cyan and pink indicate electron depletion and accumulation, respectively; isosurface value=±0.002). c) Symmetry analysis of Cu SACs and De‐sat Cu SACs. d) Electron localization function (ELF) map of De‐sat Cu SACs. Blue indicates regions of low electron localization. e) Projected density of states (PDOS) of Cu *d* orbitals in Cu SACs and De‐sat Cu SACs. f) Key adsorption intermediates in propargylic substitution. g) Optimized adsorption configurations of the deprotonated propargyl carbonate on Cu SACs and De‐sat Cu SACs.

We then performed DFT calculations to investigate how coordination de‐saturation modulates the catalytic behavior of single‐atom Cu sites. The corresponding active sites including CuN_4_ for Cu SACs and CuN_3_ for De‐sat Cu SACs were examined to explore their intrinsic structure‐activity relationships. As shown in **Figure** **5**a, removal of a coordinating N atom leads to a slight elongation of Cu–N bonds in De‐sat Cu SACs (1.952 Å in CuN_3_ versus 1.933 Å in CuN_4_), reflecting a reduced coordination and increased structural flexibility of the Cu center. The charge density difference plot (Figure 5b) reveals that CuN_4_ exhibits a symmetric electron polarization and a large Bader charge (+0.94 |e|), indicating a strong ligand field and low orbital accessibility. In contrast, CuN_3_ site shows an anisotropic charge accumulation oriented toward the open axial site, with lower Bader charge (+0.69|e|), consistent with weakened ligand donation and a more exposed, reactive Cu center.

The projected density of states (PDOS) analyses (Figure 5c,d) further reveal distinct electronic and chemical properties between CuN_4_ and CuN_3_ sites. The high D_4h_‐symmetry and near‐saturated ligand field of CuN_4_ lower its *d*‐band center (–3.25 eV), rendering the Cu center electronically inert and poorly electrophilic. Although the $d_{x^{2}-y^{2}}$ orbital lies near the Fermi level, its strong antibonding nature and in‐plane orientation preclude effective interaction with propargyl substrates approaching along the axial (z) direction. Upon symmetry reduction to C_2v_, the ligand field in CuN_3_ weakens, causing the $d_{z^{2}}$ orbital to shift closer to the Fermi level and becomes both spatially and energetically aligned with the terminal C *p* orbital of the propargyl carbonate **1**. The *d*‐band center of CuN_3_ site rises to –1.79 eV, indicating its enhanced orbital activity. Consistent with the electronic structure changes, the electron localization function (ELF) maps of CuN_3_ reveal a well‐defined electrophilic channel oriented along the defect direction (Figure 5e). Considering the potential variation in vacancy size during the etching process, we also examined another CuN_3_ model adjacent to a larger vacancy (Figure S13, Supporting Information). The charge density difference plot and ELF (Figure S14 and S15, Supporting Information) closely resemble those of the CuN_3_ model shown in Figure 5b,d. Additionally, the *d*‐band center (–1.76 eV) is nearly identical to that of CuN_3_ (Figure 5e; Figure S16, Supporting Information). These similarities suggest that the electronic properties are primarily influenced by the change in the local coordination of Cu rather than the size of adjacent vacancies.

The initially formed copper–alkynyl species serve as key adsorption intermediates that promote subsequent substitution (Figure 5f). Frontier orbital analysis of propargyl carbonate **1** (Figure S17, Supporting Information) shows the highest occupied molecular orbital (HOMO) is primarily localized on two C atoms, forming a π‐bonding orbital well‐suited for Cu coordination. Adsorption configuration analysis (Figure 5g; Figure S18, Supporting Information) confirms their enhanced stabilization on desaturated CuN_3_ sites, which bind the alkynyl intermediate more strongly than CuN_4_, as evidenced by shorter Cu─C distances (1.872 Å vs 1.937 Å) and more favorable adsorption energies (*E*_ads_ = –2.59 eV vs –2.88 eV). Additional adsorption behaviors of all reaction intermediates over CuN_3_ site are provided in Figure S19 (Supporting Information). Taken together, these results demonstrate that coordination de‐saturation of the Cu center not only modulates its local electronic properties but also enhances its electrophilicity and unlocks symmetry‐ and energy‐matched frontier orbitals that facilitate propargyl carbonates binding and activation, thereby accelerating propargylic substitution catalysis.

## 3 Conclusion

This study demonstrates a facile yet elegant top‐down de‐saturation strategy to fabricate asymmetric de‐saturated copper single‐atom catalysts via KOH‐mediated Joule thermal shock treatment. These De‐sat Cu SACs showcase remarkable performance in propargylic substitution catalysis, including enhanced mechanistic insights and exceptional substrate versatility, applicable to a wide array of nucleophiles, including N–, C–, and O–based species, as well as diverse aryl, alkyl, tertiary, and cyclic propargylic carbonates. DFT calculations confirm that the electronic structure of CuN_3_ originates specifically from the coordination‐desaturated Cu atom, where symmetry reduction elevates *d*‐orbital energy, creating an activated catalytic center that enables more efficient activation of alkynyl species than CuN_4_ in propargylic substitution reactions. Our findings open a new pathway for the rational design and synthesis of high‐performance SACs, driving challenging catalytic transformations toward sustainable fine chemical and drug manufacturing.

## 4 Experimental Section

### Materials

Deionized water was used throughout this study. Polystyrene and polyacrylonitrile were purchased from Sigma–Aldrich Company. Unless otherwise noted below, commercially available reagents were used throughout without further purification and all reactions were performed using standard Schlenk techniques under an atmosphere of argon or in glovebox. Dry solvents were purchased and stored with molecular sieves in an atmosphere of argon.

### Preparation of the PAN/PS Fibers

0.4 g of polystyrene (PS) and 0.5 g of polyacrylonitrile (PAN) were dissolved into 5 mL of N,N‐dimethylformamide (DMF) under vigorous stirring at 60 °C overnight. The mixture solution was loaded into a 5 mL syringe. The voltage, feed rate, and distance between the collector and the stainless‐steel needle was carried out with 17 kV, 1 mL h^−1^, and 15 cm, respectively.

### Preparation of the PAN/PS‐Cu Fibers

In a typical synthesis process, 0.5 g of *PAN/PS fibers* and 1 g of CuCl_2_ were dispersed into 50 mL of methanol with stirring for 12 h at 60 °C. The resultant PAN/PS‐Cu^2+^ product was collected by centrifugation and washed with methanol several times, and then was dried under vacuum at 65 °C for further use.

### Preparation of the Cu SACs

The PAN/PS‐Cu*^2+^* fibers were annealed in N_2_ at 700 ^°^C for 1.5 h with a heating rate of 5 °C min^−1^ to obtain Cu SACs.

### Preparation of the CMFs

The PAN/PS fibers were annealed in N_2_ at 1000 ^°^C for 1.5 h with a heating rate of 5 °C min^−1^ to obtain CMFs.

### Preparation of the De‐sat Cu SACs

The as‐obtained Cu SACs and KOH are mixed in a 1:1 mass fraction and then placed into a tablet press for tabletting. Then, the tablets were put a commercial joule‐heating derive to yield De‐sat Cu SACs.

### Preparation of the Cu NPs@CMFs Fibers

The PAN/PS‐Cu*^2+^* fibers were annealed in N_2_ at 1000 ^°^C for 5 h with a heating rate of 5 °C min^−1^ to obtain Cu NPs@CMFs.

### Catalytic Evaluation

A typical experimental procedure for the preparation of N‐(1‐phenyl‐2‐propynyl)aniline (**3**) is described below. De‐sat Cu SACs‐12s (1.02·wt.%, 6.3 mg, 1.0 mmol%), *tert*‐butyl (1‐phenylprop‐2‐yn‐1‐yl) carbonate **1** (23.2 mg, 0.1 mmol), aniline (18.6 mg, 0.2 mmol) and Et_3_N (20.2 mg, 0.2 mmol) were placed in a 10 mL bottle and the reaction was carried out under an argon atmosphere in a glovebox. Then, MeOH (0.5 mL) was added, and the mixture was stirred at 60 ^°^C for 24 h. The mixture was concentrated under reduced pressure and the residue was purified by silica gel chromatography with n‐hexane and EtOAc (n‐hexane/EtOAc = 10/1‐6/1) as eluent to give **3** as a yellow oil (17.0 mg, 82% yield). The synthesis of other products (**4**–**51**) was also carried out using this method or a similar approach.

### Characterization

TEM images were recorded on a Hitachi‐7650 worked at 100 kV. The high‐resolution TEM, HAADF‐STEM images and corresponding Electron energy‐loss spectroscopy were recorded on a FEI Tecnai G2 F20 S‐Twin high‐resolution transmission electron microscope worked at 200 kV and a JEOL JEM‐ARM200F TEM/STEM with a spherical aberration corrector worked at 300 kV. Through‐focal HAADF series were acquired at nanometer intervals, with the first image under‐focused (beyond the beam exit surface) and the final image over‐focused (before the beam entrance surface). Flash column chromatography was performed using 200‐300 mesh silica gel. Melting points were measured on a RY‐I apparatus and uncorrected. ^1^H, ^13^C, and ^19^F NMR spectra were recorded on Varian (400 MHz) or Agilent (400or 600 MHz) spectrometers.

Chemical shifts were reported in parts per million (ppm) and refer to the appropriate residual solvent peak: ^1^H NMR were referenced to the central peak of CDCl_3_ (7.260 ppm); DMSO‐*d*_6_ (3.250 ppm); or to the internal standard TMS (0.000 ppm); ^13^C NMR were referenced to the central peak of 77.00 ppm for CDCl_3_; 39.99 ppm for DMSO‐*d*_6_. Single crystal X‐ray diffraction data was collected on a Bruker D_8_ Venture diffractometer at 293(2) K or 173(0) K. Using Olex_2_, the structure was solved with the SHELXT structure solution program using Intrinsic Phasing and refined with the SHELXL refinement package using Least Squares minimisation. Powder X‐ray diffraction (XRD) measurements were recorded on a Rigaku Miniflex‐600 operated at 40 kV voltage and 15 mA current using a Cu K_α_ radiation (λ = 0.15406 nm) at a step width of 8°·min^−1^.

## Conflict of Interest

The authors declare no conflict of interest.

## Supporting information



Supporting Information

## Data Availability

The data that support the findings of this study are available in the supplementary material of this article.
