# Supplementary material for: De‐Saturation of Single‐Atom Copper Catalysts for Accelerating Propargylic Substitution Reactions
Source: Adv Mater. 2025 Aug 25;37(50):e09221. doi: 10.1002/adma.202509221 (PMC12710579; doi:10.1002/adma.202509221)
Supplement: Supplementary file 1 — Supporting Information [file ADMA-37-e09221-s001.docx]

Supporting Information *for*

De-Saturation of Single-Atom Copper Catalysts for Accelerating Propargylic Substitution Reactions

Qilong Cai^1,†^, Yang Meng^1,2†^, Chao Wu^4,†^, Wenjia Qu^1^, Qiang, Wang^1^, Tan Li^1^, Chengyi Liu^3^ Jinxing Chen^1^, Huihui, Lin^1^, Qian He^5^, Yafei Zhao^1,6^*, Shibo Xi^3^,*, Jiong Lu^1,2^*

^1^Department of Chemistry, National University of Singapore, Singapore, Singapore.

^2^National University of Singapore (Suzhou) Research Institute, No. 377 Linquan Street, Suzhou, Jiangsu, P. R. China.

^3^Institute of Sustainability for Chemicals, Energy and Environment (ISCE2), Agency for Science, Technology and Research (A*STAR), 1 Pesek Road Jurong Island, Singapore, Singapore.

^4^College of Materials Science and Engineering, Sichuan University, Chengdu 610065, P. R. China.

^5^Department of Materials Science and Engineering, National University of Singapore, Singapore, Singapore.

^6^State Key Laboratory of Precision and Intelligent Chemistry, University of Science and Technology of China (USTC), Jinzhai Road 96, Hefei, Anhui 230026, P. R. China.

[†] These authors contributed equally to this work.

*E-mail: [yafeizhao@ustc.edu.cn,](mailto:yafeizhao@ustc.edu.cn,) [xi_shibo@partner.nus.edu.sg](mailto:xi_shibo@partner.nus.edu.sg), [chmluj@nus.edu.sg](mailto:chmluj@nus.edu.sg)

1. Experimental details

*Materials.* Deionized water was used throughout this study. Polystyrene and polyacrylonitrile were purchased from Sigma-Aldrich Company. Unless otherwise noted below, commercially available reagents were used throughout without further purification and all reactions were performed using standard Schlenk techniques under an atmosphere of argon or in glovebox. Dry solvents were purchased and stored with molecular sieves in an atmosphere of argon.

*Preparation of the PAN/PS fibers.* 0.4 g of polystyrene (PS) and 0.5 g of polyacrylonitrile (PAN) were dissolved into 5 mL of N,N-dimethylformamide (DMF) under vigorous stirring at 60 °C overnight. The mixture solution was loaded into a 5 mL syringe. The voltage, feed rate, and distance between the collector and the stainless-steel needle was carried out with 17 kV, 1 mL h^–1^, and 15 cm, respectively.

*Preparation of the PAN/PS-Cu fibers.* In a typical synthesis process, 0.5 g of *PAN/PS fibers* and 1 g of CuCl_2_ were dispersed into 50 mL of methanol with stirring for 12 h at 60 ºC. The resultant PAN/PS-Cu^2+^ product was collected by centrifugation and washed with methanol several times, and then was dried under vacuum at 65 °C for further use.

*Preparation of the Cu SACs.*The PAN/PS-Cu*^2+^* fibers were annealed in N_2_ at 700 ^o^C for 1.5 h with a heating rate of 5 °C min^–1^ to obtain Cu SACs.

*Preparation of the CMFs.*The PAN/PS fibers were annealed in N_2_ at 1000 ^o^C for 1.5 h with a heating rate of 5 °C min^–1^ to obtain CMFs.

*Preparation of the De-sat Cu SACs.* The as-obtained Cu SACs and KOH are mixed in a 1:1 mass fraction and then placed into a tablet press for tabletting. Then, the tablets were put a commercial joule-heating derive to yield De-sat Cu SACs.

*Preparation of the Cu NPs@CMFs fibers.* The PAN/PS-Cu*^2+^* fibers were annealed in N_2_ at 1000 ^o^C for 5 h with a heating rate of 5 °C min^–1^ to obtain Cu NPs@CMFs.

*Characterization.* TEM images were recorded on a Hitachi-7650 worked at 100 kV. The high-resolution TEM, HAADF-STEM images and corresponding Electron energy-loss spectroscopy were recorded on a FEI Tecnai G2 F20 S-Twin high-resolution transmission electron microscope worked at 200 kV and a JEOL JEM-ARM200F TEM/STEM with a spherical aberration corrector worked at 300 kV. Through-focal HAADF series were acquired at nanometer intervals, with the first image under-focused (beyond the beam exit surface) and the final image over-focused (before the beam entrance surface). Flash column chromatography was performed using 200-300 mesh silica gel. Melting points were measured on a RY-I apparatus and uncorrected. ^1^H, ^13^C and ^19^F NMR spectra were recorded on Varian (400 MHz) or Agilent (400 MHz or 600 MHz) spectrometers.

Chemical shifts were reported in parts per million (ppm) and refer to the appropriate residual solvent peak: ^1^H NMR were referenced to the central peak of CDCl_3_ (7.260 ppm); DMSO-*d*_6_ (3.250 ppm); or to the internal standard TMS (0.000 ppm); ^13^C NMR were referenced to the central peak of 77.00 ppm for CDCl_3_; 39.99 ppm for DMSO-*d*_6_. Single crystal X-ray diffraction data was collected on Bruker D_8_ Venture diffractometer at 293(2) K or 173(0) K. Using Olex_2_, the structure was solved with the SHELXT structure solution program using Intrinsic Phasing and refined with the SHELXL refinement package using Least Squares minimisation. Powder X-ray diffraction (XRD) measurements were recorded on a Rigaku Miniflex-600 operated at 40 kV voltage and 15 mA current using a Cu K_α_ radiation (λ=0.15406 nm) at a step width of 8°·min^–1^.

The XANES and EXAFS measurements were carried out at the XAFCA beamline of the Singapore Synchrotron Light Source (SSLS)40. A Si(111) double-crystal monochromator was used to filter the X-ray beam. Copper foil was used for the energy calibration and all samples were measured under transmission mode. The acquired EXAFS data were extracted and processed according to the standard procedures using the ATHENA module implemented in the IFEFFIT software packages.^1–4^ The *k*^2^-weighted EXAFS spectra were obtained by subtracting the post-edge background from the overall absorption and then normalizing with respect to the edge-jump step. Subsequently, *k*^2^-weighted χ(*k*) data in the k-space were Fourier transformed to real (R) space to separate the EXAFS contributions from different coordination shells. To obtain the quantitative structural parameters around central atoms, least-squares curve parameter fitting was performed using the ARTEMIS module of IFEFFIT software packages. EXAFS spectra were analyzed using the following equation:

$$\text{χ}\text{(k)=}\sum_{\text{j}} \frac{\text{N}_{\text{j}}\text{S}_{0}^{2}\text{F}_{\text{j}}\left( \text{k} \right)}{\text{K}{\text{R}_{\text{j}}}^{2}}\text{exp}\left[ \text{-2}\text{K}^{2}\text{σ}_{\text{j}}^{2} \right]\text{exp}\left[ \frac{\text{-2}\text{R}_{\text{j}}}{\text{λ}\left( \text{k} \right)} \right]\text{Sin}\left[ \text{2k}\text{R}_{\text{j}}\text{+}\text{Φ}_{\text{j}}\left( \text{k} \right) \right]$$

S_0_^2^ is the amplitude reduction factor, F_j_(k) is the effective curved-wave back scattering amplitude, N_j_ is the number of neighbors in the j_th_ atomic shell, R_j_ is the distance between the X-ray absorbing central atom and the atoms in the j th atomic shell (back scatterer), λ is the mean free path in Å, ϕ_j_(k) is the phase shift (including the phase shift for each shell and the total central atom phase shift), σ_j_ is the Debye-Waller parameter of the j_th_ atomic shell (variation of distances around the average R_j_). The functions F_j_(k), λ and ϕ_j_(k) were calculated with the ab initio code FEFF8.2.^5–7^

*Catalytic evaluation**.* A typical experimental procedure for the preparation of N-(1-phenyl-2-propynyl)aniline (**3**) is described below. De-sat Cu SACs-12s (1.02·wt%, 6.3 mg, 1.0 mmol%), *tert*-butyl (1-phenylprop-2-yn-1-yl) carbonate **1** (23.2 mg, 0.1 mmol), aniline (18.6 mg, 0.2 mmol) and Et_3_N (20.2 mg, 0.2 mmol) were placed in a 10 mL bottle and the reaction was carried out under an argon atmosphere in a glovebox. Then, MeOH (0.5 mL) was added, and the mixture was stirred at 60 ^o^C for 24 h. The mixture was concentrated under reduced pressure and the residue was purified by silica gel chromatography with n-hexane and EtOAc (n-hexane/EtOAc=10/1-6/1) as eluent to give **3** as a yellow oil (17.0 mg, 82% yield). The synthesis of other products (**4**–**51**) was also carried out using this method or a similar approach.

*Computational methods.* The periodic density function theory calculations of geometric and electronic properties were performed using the Vienna ab initio Simulation Package (VASP).^8^ The generalized gradient approximation (GGA) with the PBE functional was employed to describe the exchange−correlation potential.^9^ The electron−ion interaction was described by the projector augmented wave (PAW) method with a cutoff energy of 520 eV for the plane-wave basis sets. The van der Waals interaction was accounted for using the dispersion correction of Grimme’s method (DFT-D3).^10,11^ The conjugated gradient method with a converging tolerance of 0.01 eV/Å for the force on each atom and 3×3×1 Monkhorst−Pack grid was used for the geometry optimization. A vacuum layer of 15 Å was used to avoid artificial interlayer interaction. The molecular DFT calculations were carried out using Gaussian16.6-31G(d) Gaussian basis sets were used for the C, N, O and H atoms.^12^ The effective core potential of LANL2DZ was adopted for Cu atom. The adsorption energy (*E*_ads_) of an adsorbate (X) on the adsorbent CuN_n_ was defined by

$E_{\text{ads}}=E_{\text{CuN}_{\text{n}}+\text{X}}-E_{\text{CuN}_{\text{n}}}-E_{\text{X}}$

where $E_{\text{CuN}_{\text{n}}+\text{X}}$, $E_{\text{CuN}_{\text{n}}}$and *E*_X_ represent the total energy of the adsorption system, substrate, and free X species, respectively.


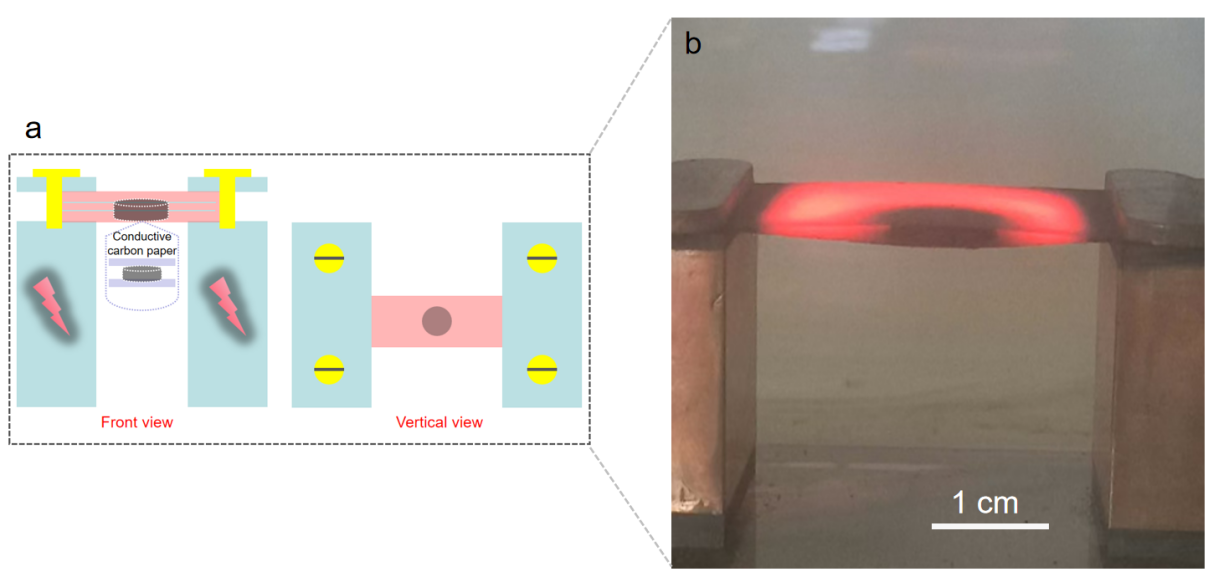


**Figure S1.** (a) Schematic diagram to illustrate the working condition of Joule heating device (b) Photograph of catalysts at high temperature.


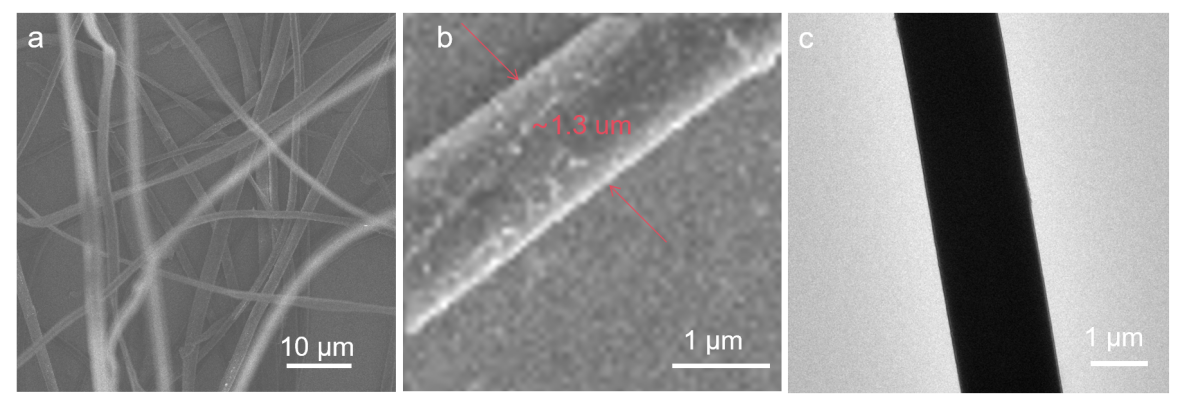


**Figure S2.** (a) FESEM (b) Magnified FESEM images and (c) TEM image of PAN/PS fibers.


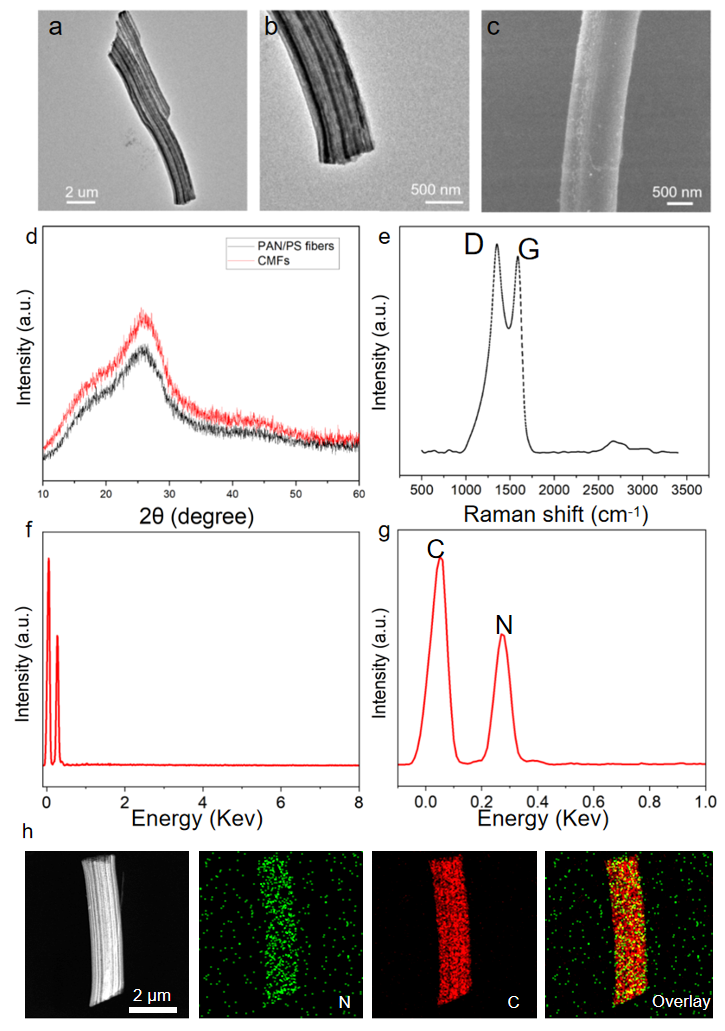


**Figure S3.** (a,b) TEM images, (c) FESEM image, and (d) XRD pattern of CMFs and CMFs. (e) Raman spectrum and (f,g) EDX spectra of CMFs are shown in a wide energy range (f) and small energy range (g). (h) HAADF-STEM and the corresponding elemental mapping images of CMFs.

HAADF-STEM and corresponding elemental mapping images confirm the uniform distribution of C and N elements within individual CMFs. The C and N content in CMFs is determined to be approximately 98.28% and 1.72%, respectively.


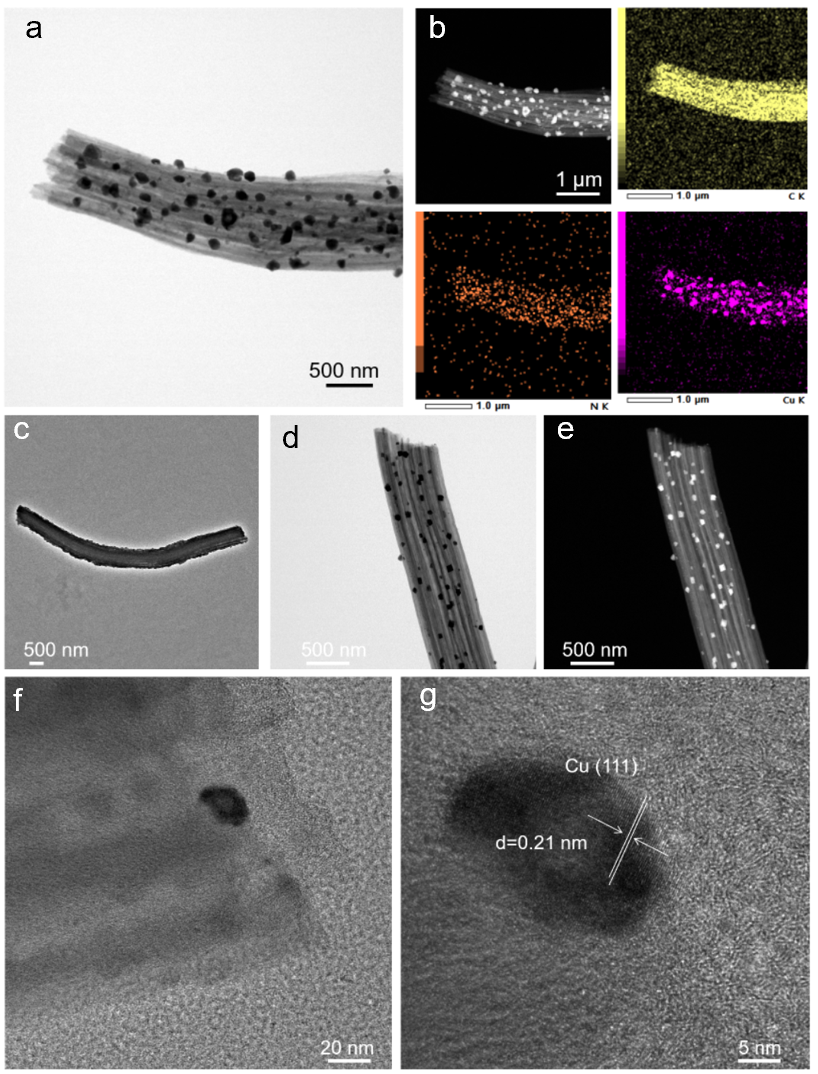


**Figure S4.** (a) HAADF-STEM (b) the corresponding elemental mapping images (c) TEM (d,e) HAADF-STEM images and (f,g) HRTEM images of Cu NPs@CMFs.


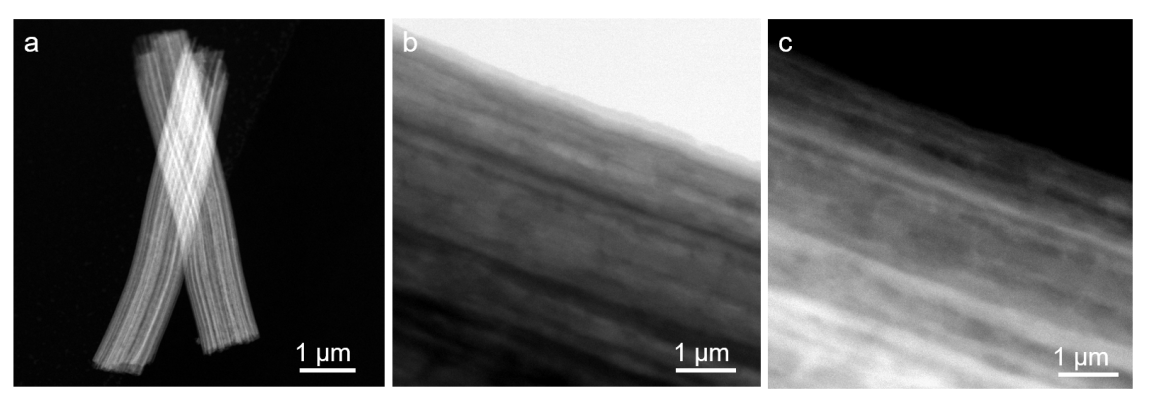


**Figure S5.** HAADF-STEM and TEM images of De-sat Cu SACs-12s.


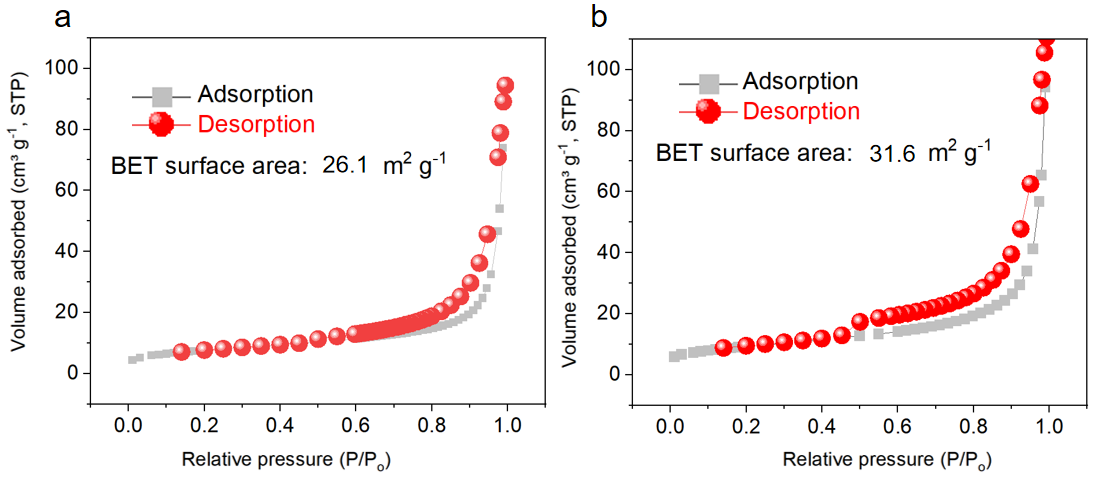


**Figure S6.** The isothermal N_2_ adsorption-desorption curves and BET surface area of (a) Cu SACs and (b) De-sat Cu SACs-12s.


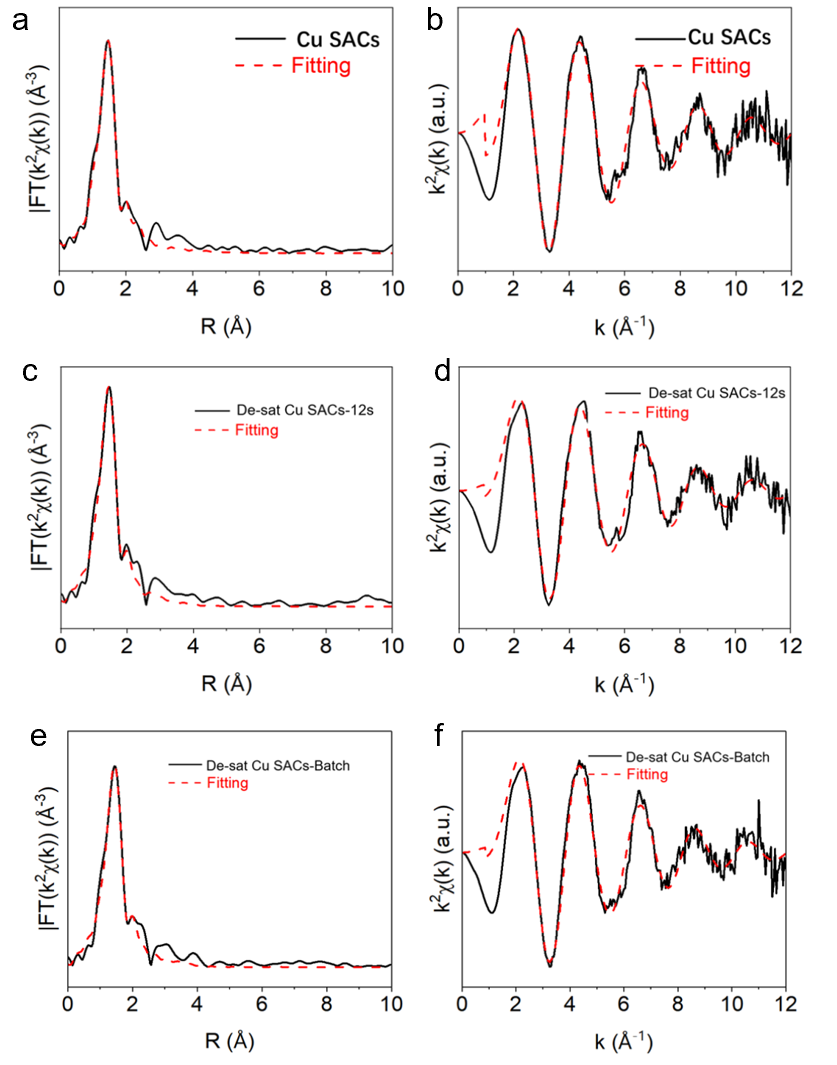


**Figure S7.** EXAFS fitting results of the FT EXAFS space and the k 2χ(k) oscillation space for the Cu SACs (a,b) De-sat Cu SACs-12s (c,d) and De-sat Cu SACs-Batch (Batch production, also etched for 12 seconds) (e,f).


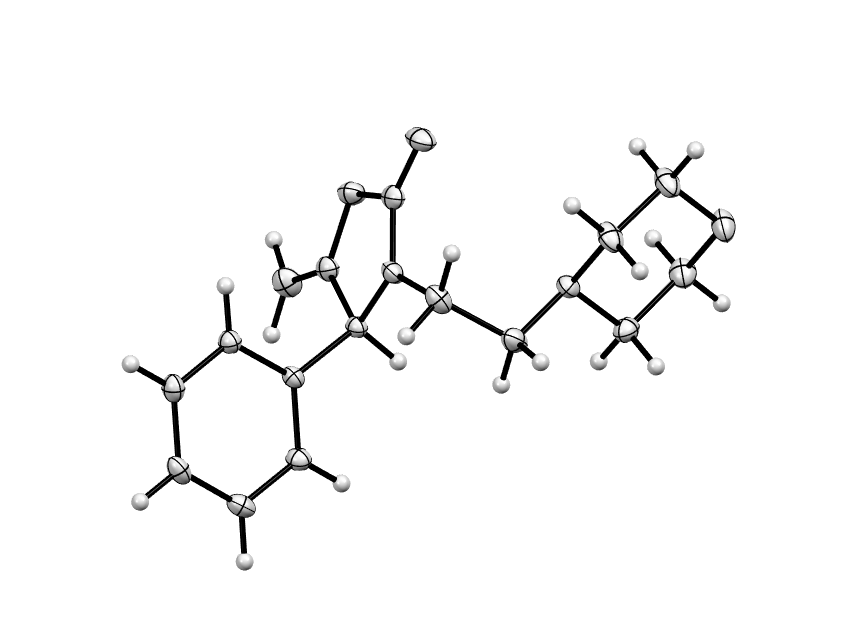


**Figure S8.** X-ray structure of **31**.

X-ray crystallographic data for 31: crystals suitable for X-ray single-crystal diffraction analysis was obtained through slowly evaporating the mixture solution of DCM and n-hexane at room temperature. CIF file for complex 31 has been deposited at the Cambridge Crystallographic Data Centre with deposition number 2447422. Copies of these data can be obtained, free of charge, on application to the CCDC, 12 Union Road, Cambridge CB2 1EZ, UK [fax: +44(1223)336033; e-mail: deposit@ccdc.cam.ac.uk].

**Figure S9.** Examples show the limitations of the substrate scopes.

These results indicate that strongly coordinating groups (–COOH, –SH) can partially or substantially inhibit the reaction, likely by binding to the metal center and disrupting the catalytic cycle. In addition, the reaction does not proceed with internal propargylic ester. These substrates represent limitations of the current method.


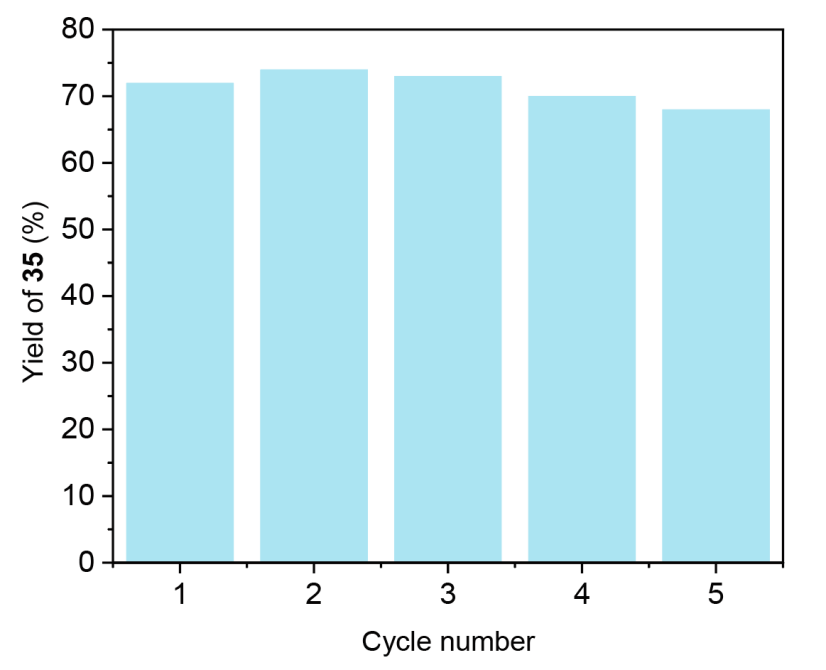


**Figure S10.** Cycling test for De-sat Cu SACs-12s in the propargyl substitution reaction.

Reaction conditions: propargyl carbonates **34** (1 mmol), PhNH_2_ (2 mmol), Et_3_N (2 mmol), De-sat Cu SACs-12s (2.0 mmol%), MeOH (5 mL), 60^o^C, 24 h. NMR yield using CH_2_Br_2_ (100 uL) as internal standard.

Catalyst recovery procedure: After the reaction was complete, 5 mL of DCM was added to the mixture, which was then subjected to centrifugation. The catalyst was recovered by filtration using 0.1 mm filter and washed sequentially with n-hexane and DCM. The catalyst was then air-dried prior to use in subsequent reaction cycles.


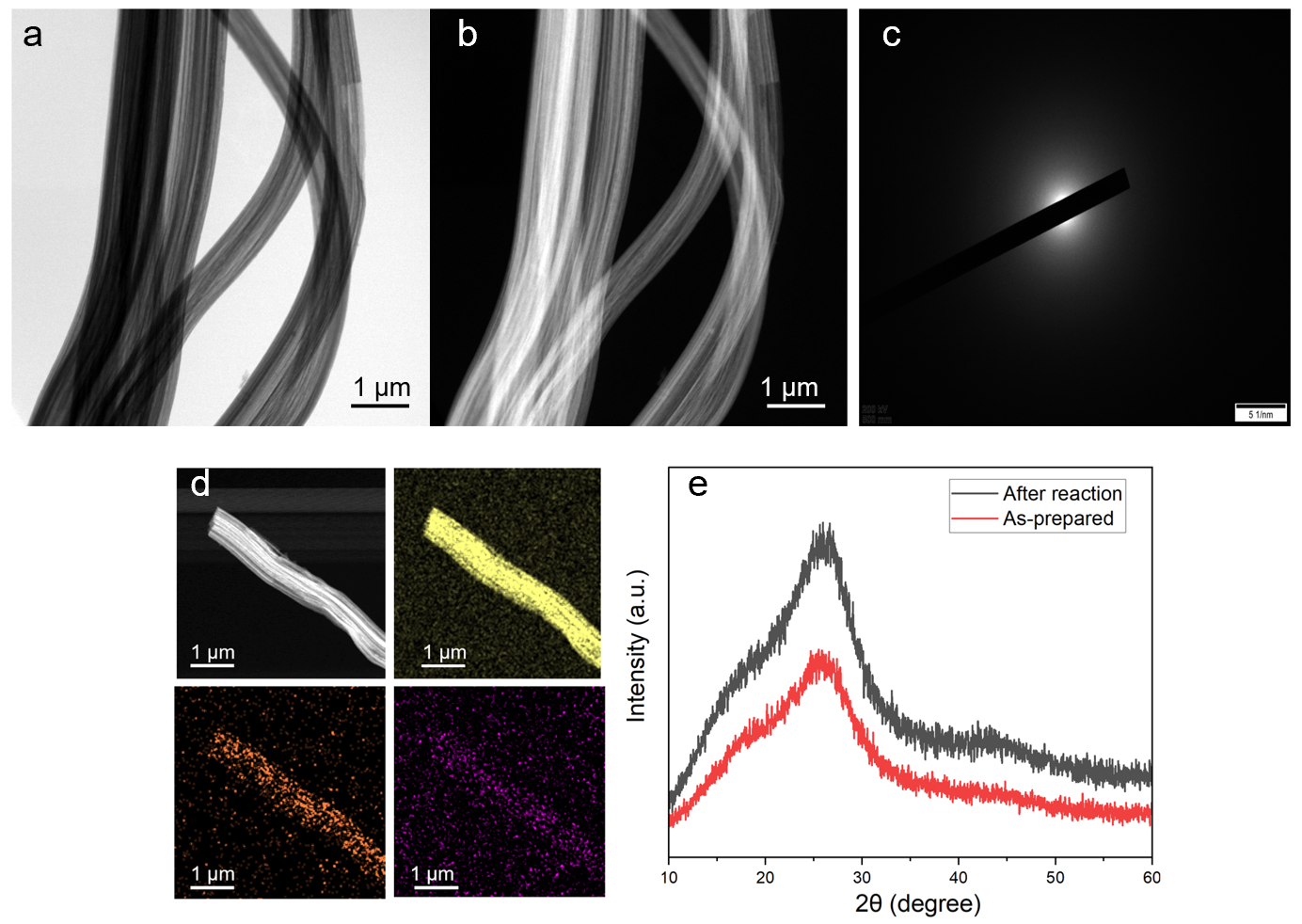


**Figure S11.** (a) TEM (b) HAADF-STEM (c) Selected area electron diffraction images (d) HAADF-STEM and the corresponding elemental mapping images and (e) XRD pattern of De-sat Cu SACs-12s before and after reaction.

The De-sat Cu SACs-12s catalyst still retains the original fiber-like structure after reaction, indicating that the matrix can withstand the harsh testing conditions.^13^

Elemental mappings (Figure S10d) of a single De-sat Cu SACs-12s demonstrate that the C, N and Cu elements are uniformly distributed throughout the entire fiber after the reaction.^14,15^ Moreover, the absence of Cu nanoparticles related peaks (Figure S10e) indicates no significant agglomeration of Cu species.^16^

**Figure S12.** X-ray structure of **51**.

X-ray crystallographic data for 51: crystals suitable for X-ray single-crystal diffraction analysis was obtained through slowly evaporating the mixture solution of DCM and n-hexane at room temperature. CIF file for complex 51 has been deposited at the Cambridge Crystallographic Data Centre with deposition number 2445459. Copies of these data can be obtained, free of charge, on application to the CCDC, 12 Union Road, Cambridge CB2 1EZ, UK [fax: +44(1223)336033; e-mail: deposit@ccdc.cam.ac.uk].


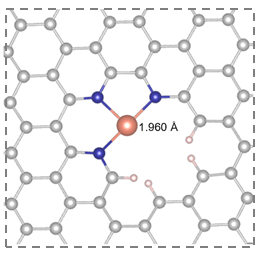


**Figure S13.** Theoretical model of CuN_3_ with an adjacent large vacancy site.


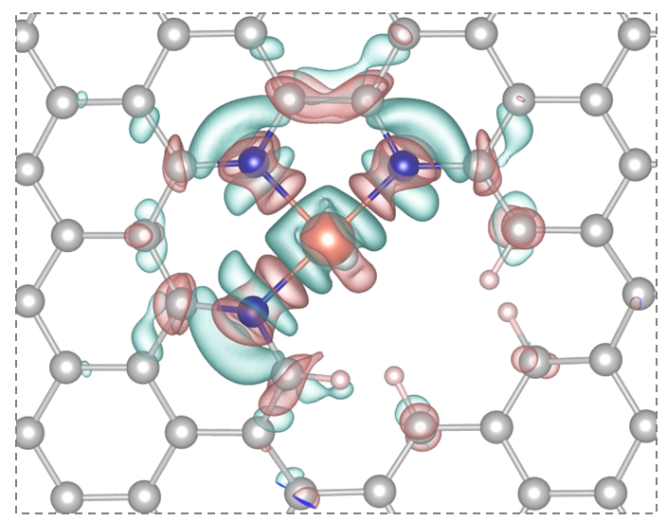


**Figure S14.** Charge density difference plot of CuN_3_ adjacent to a large vacancy site.


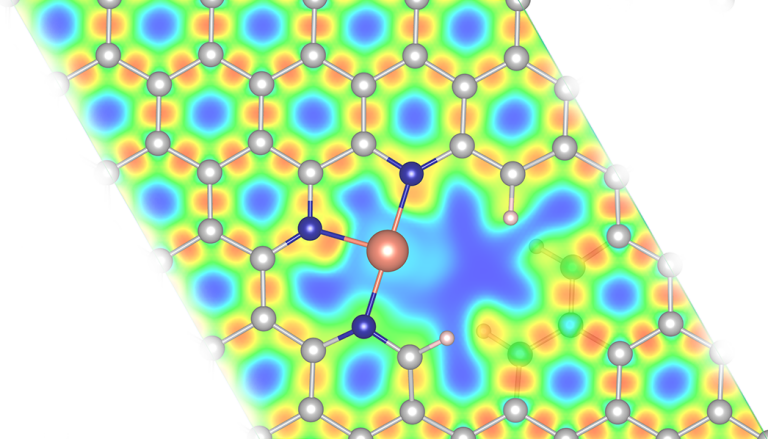


**Figure S15.** ELF map of CuN_3_ adjacent to a large vacancy site.


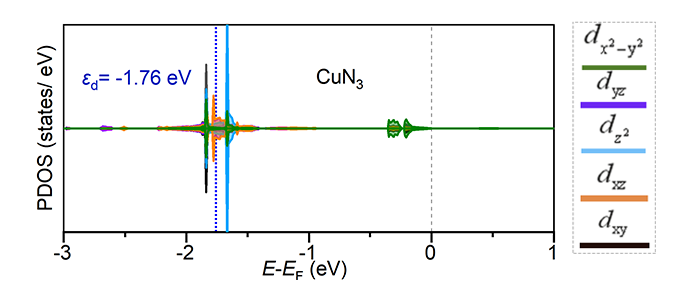


**Figure S16.** Projected density of states (PDOS) of Cu *d* orbitals in CuN_3_ adjacent to a large vacancy site.


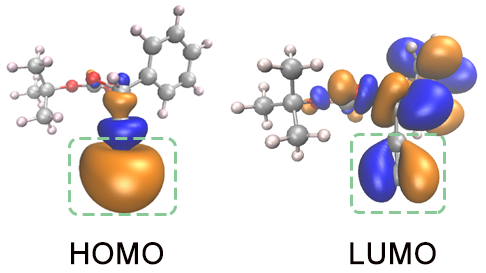


**Figure S17.** Frontier molecular orbitals of propargyl carbonate **1**.


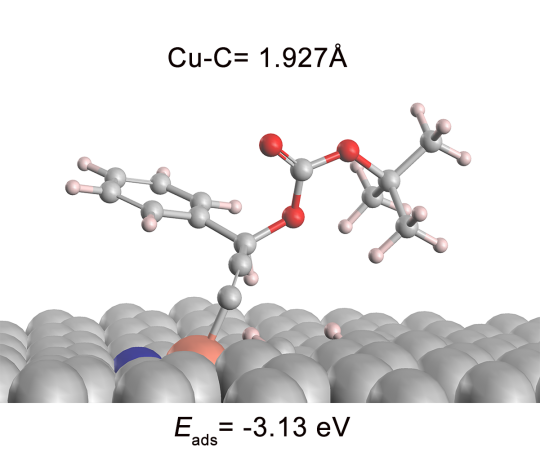


**Figure S18.** Optimized adsorption configurations of the propargyl carbonate **1** on CuN_3_ adjacent to a large vacancy site.


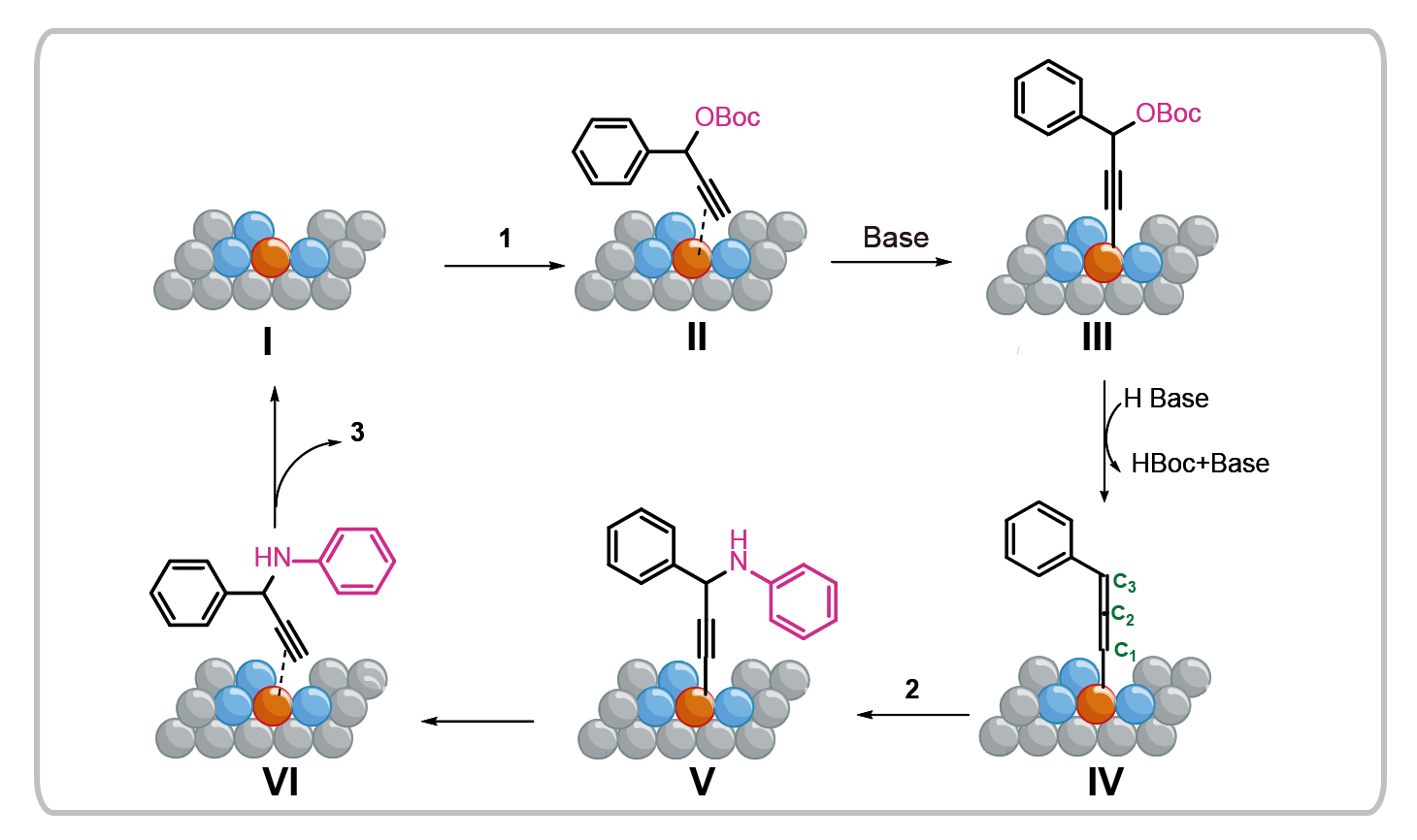


**Figure S19.** A schematic diagram illustrating the proposed mechanism for the propargylic substitution reaction catalyzed by de-saturation CuN_3_ (Gray: C atom, blue: N atom, red: Cu atom).

Based on integrated experimental and computational evidence, we propose a plausible reaction mechanism. The catalytic cycle initiates at the desaturated CuN_3_ single-atom site (**I**), which adsorbs propargylic substrate **1** to form surface-bound intermediate **II**. Subsequent base-mediated deprotonation generates an alkynyl anion that coordinates to the Cu center, affording Cu-alkynyl complex **III**. This is followed by Cu-promoted elimination of tert-butyl carbonate (-OBoc) group from the intermediate **III** and generate the key allenyl-copper species **IV**. Nucleophilic attack by aniline at the (C_3_) position of **IV** then forms intermediate **V**, whose protonation delivers propargylic amine **3** as the final product with concomitant regeneration of the CuN_3_ catalyst **I**. This mechanistic framework elucidates the stepwise functions of CuN_3_ in substrate activation, intermediate stabilization, and catalytic turnover.

**Table S1**. Inductively coupled plasma optical emission spectrometry (ICP-OES) measurements.

| Sample | Metal content of Cu (wt%) |
| --- | --- |
| Cu SACs | 1.17 |
| De-sat Cu SACs-6s | 1.15 |
| De-sat Cu SACs-12s | 1.02 |
| De-sat Cu SACs-18s | 0.84 |
| De-sat Cu SACs-24s | 0.62 |
| De-sat Cu SACs-30s | 0.31 |
| De-sat Cu SACs-36s | 0.15 |

**Table S2.** EXAFS fitting parameters of Cu *K*-edge for Cu samples.

| Sample | Path | CN | R(Å) | σ^2^(10^-3^Å^2^) | ΔE_0_(eV) | R factor |
| --- | --- | --- | --- | --- | --- | --- |
| Cu SACs | Cu-N | 4.0 | 1.93 | 4.1 | 6.0 | 0.004 |
| De-sat Cu SACs | Cu-N | 3.3 | 1.92 | 7.3 | 3.3 | 0.005 |
| Cu SACs-Batch | Cu-N | 3.4 | 1.93 | 7.6 | 3.1 | 0.004 |

CN is the coordination number; R is the interatomic distance (the bond length between central atoms and surrounding coordination atoms); σ^2^ is the Debye-Waller factor (a measure of thermal and static disorder in absorber-scatterer distances); ΔE_0_ is edge-energy shift (the difference between the zero kinetic energy value of the sample and that of the theoretical model). Error bounds that characterize the structural parameters obtained by EXAFS spectroscopy were estimated as CN ± 20%; R ± 2%; σ^2^ ± 20%; ΔE_0_ ± 20%.

**Table S3.** Crystal data and structure refinement for **31**

Identification code **31**

Empirical formula C_16_H_20_N_2_O_3_

Formula weight 288.34

Temperature 100.00 K

Wavelength 0.71073 Å

Crystal system Triclinic

Space group P1

Unit cell dimensions a = 6.0112(4) Å a= 77.978(3)°.

b = 8.1227(6) Å b= 72.934(3)°.

c = 8.1312(6) Å g = 89.791(3)°.

Volume 370.52(5) Å^3^

Z 1

Density (calculated) 1.292 Mg/m^3^

Absorption coefficient 0.090 mm^-1^

F(000) 154

Crystal size 0.257 x 0.188 x 0.138 mm^3^

Theta range for data collection 2.569 to 30.520°.

Index ranges -8<=h<=8, -11<=k<=11, -11<=l<=11

Reflections collected 20022

Independent reflections 4540 [R(int) = 0.0324]

Completeness to theta = 25.242° 100.0 %

Absorption correction Semi-empirical from equivalents

Max. and min. transmission 0.7461 and 0.7231

Refinement method Full-matrix least-squares on F^2^

Data / restraints / parameters 4540 / 3 / 190

Goodness-of-fit on F^2^ 1.069

Final R indices [I>2sigma(I)] R1 = 0.0321, wR2 = 0.0866

R indices (all data) R1 = 0.0334, wR2 = 0.0877

Absolute structure parameter -0.4(3)

Extinction coefficient n/a

Largest diff. peak and hole 0.266 and -0.191 e.Å^-3^

**Table S4**. Comparison of the catalytic performance.

| A | P | [Cu] (1.0 mol%) | Yield (%) |
| --- | --- | --- | --- |
|  |  | Cu SACs | 3 |
|  |  | De-sat Cu SACs | 82 |
|  |  | Cu SACs | 18 |
|  |  | De-sat Cu SACs | 78 |
|  |  | Cu SACs | 1 |
|  |  | De-sat Cu SACs | 63 |

Reaction conditions : propargyl carbonates (0.1 mmol), PhNH_2_ (0.2 mmol), Et_3_N (0.2 mmol), De-sat Cu SACs-12s / Cu SACs (1.0 mmol%), MeOH (0.5 mL), 60 ^o^C, 24 h. NMR yield using CH_2_Br_2_ (20 uL) as internal standard.

**Table S5**. Comparison of the catalytic performance.

| **[Cu] (1.0 mol%)** | **Yield of 22 (%)** |
| --- | --- |
| De-sat Cu SACs | 67 |
| Cu SACs | 20 |
| Cu-PC | 0 |
| CuI | 0 |

Reaction conditions : propargyl carbonates (0.1 mmol), cyclohexylamine (0.2 mmol), Et_3_N (0.2 mmol), [Cu] (1.0 mmol%), MeOH (0.5 mL), 60 ^o^C, 24 h. NMR yield using CH_2_Br_2_ (20 uL) as internal standard.

**Table S6**. Comparison of the kinetic profiles.

| **Time/ h** | **De-sat Cu SACs-12s** Yield (%) | **Cu SACs**  Yield (%) |
| --- | --- | --- |
| 1 | 12 | 1.4 |
| 2 | 19 | 1.7 |
| 4 | 29 | 2.3 |
| 8 | 45 | 3.3 |
| 24 | 72 | 8.4 |

Reaction conditions : propargyl carbonates **34** (0.1 mmol), PhNH_2_ (0.2 mmol), Et_3_N (0.2 mmol), De-sat Cu SACs-12s (1.0 mmol%), MeOH (0.5 mL), 60 ^o^C, x h. GC yield using tridecane (20 uL) as internal standard.

**Table S7.** Crystal data and structure refinement for **51**

Identification code **51**

Empirical formula C_19_H_21_NO_2_

Formula weight 295.37

Temperature 170.00 K

Wavelength 1.34139 Å

Crystal system Orthorhombic

Space group P2**_1_**2**_1_**2**_1_**

Unit cell dimensions

a = 11.4701(2) Å a= 90°.

b = 11.7714(3) Å b= 90°.

c = 23.6821(5) Å g = 90°.

Volume 3197.54(12) Å3

Z 8

Density (calculated) 1.227 Mg/m^3^

Absorption coefficient 0.399 mm^-1^

F(000) 1264

Crystal size 0.17 x 0.17 x 0.05 mm3

Theta range for data collection 3.247 to 54.937°.

Index ranges -8<=h<=13, -14<=k<=13, -28<=l<=27

Reflections collected 35355

Independent reflections 6004 [R(int) = 0.0489]

Completeness to theta = 53.594° 99.6 %

Absorption correction Semi-empirical from equivalents

Max. and min. transmission 0.7508 and 0.6354

Refinement method Full-matrix least-squares on F2

Data / restraints / parameters 6004 / 0 / 407

Goodness-of-fit on F2 1.032

Final R indices [I>2sigma(I)] R1=0.0320, wR2=0.0840

R indices (all data) R1=0.0329, wR2=0.0847

Absolute structure parameter 0.10(7)

Extinction coefficient n/a

Largest diff. peak and hole 0.538 and -0.138 e.Å-3

Absorption coefficient 3.662 mm^-1^

F(000) 32

Crystal size 0.16 x 0.05 x 0.01 mm^3^

Theta range for data collection 0.828 to 27.510°.

Index ranges -31<=h<=26, -31<=k<=31, -12<=l<=12

Reflections collected 48096

Independent reflections 12859 [R(int) = 0.0694]

Completeness to theta = 25.242° 100.0 %

Absorption correction Semi-empirical from equivalents

Max. and min. transmission 0.7456 and 0.6094

Refinement method FμLl-matrix least-squares on F^2^

Data / restraints / parameters 12859 / 1 / 661

Goodness-of-fit on F^2^ 1.032

Final R indices [I>2sigma(I)] R1 = 0.0444, wR2 = 0.0660

R indices (all data) R1 = 0.1203, wR2 = 0.0819

Absolute structure parameter 0.02(2)

Extinction coefficient n/a

Largest diff. peak and hole 0.618 and -0.501 e.Å^-3^

**2.Substrate scope**

General Procedure: A typical experimental procedure for the preparation of *N*-(1-phenyl-2-propynyl)aniline (**3**) is described below. De-sat Cu SACs-12s (1.02·wt%, 6.3 mg, 1.0 mmol%), tert-butyl (1-phenylprop-2-yn-1-yl) carbonate **1** (23.2 mg, 0.1 mmol), aniline (18.6 mg, 0.2 mmol) and Et_3_N (20.2 mg, 0.2 mmol) were placed in a 10 mL bottle and the reaction was carried out under an argon atmosphere in a glovebox. Then, MeOH (0.5 mL) was added, and the mixture was stirred at 60 ^o^C for 24 h. The mixture was concentrated under reduced pressure and the residue was purified by silica gel chromatography with n-hexane and EtOAc (n-hexane/EtOAc = 10/1-6/1) as eluent to give **3** as a yellow oil (17.0 mg, 82% yield).

Spectroscopic data of **3**-**51** are as follows.

***N*-(1-phenyl-2-propynyl)aniline (3)**^17^

Yellow oil, 17.0 mg, 82% yield. ^1^H NMR (400 MHz, CDCl_3_) δ 7.60 (d, *J* = 6.8 Hz, 2H), 7.43–7.32 (m, 3H), 7.21 (t, *J* = 7.7 Hz, 2H), 6.79 (t, *J* = 7.3 Hz, 1H), 6.73 (d, *J* = 8.0 Hz, 2H), 5.32 (d, *J* = 4.4 Hz, 1H), 4.04 (br, 1H), 2.47 (d, *J* = 2.2 Hz, 1H) ppm.

**4-methoxy-*N*-(1-phenylprop-2-yn-1-yl)aniline (4)**^17^

Yellow oil, 20.2 mg, 85% yield. ^1^H NMR (400 MHz, CDCl_3_) δ 7.56 – 7.49 (m, 2H), 7.35 – 7.22 (m, 3H), 6.76 – 6.69 (m, 2H), 6.68 – 6.59 (m, 2H), 5.13 (d, *J* = 2.2 Hz, 1H), 3.67 (s, 3H), 2.39 (d, *J* = 2.3 Hz, 1H) ppm.

**4-chloro-*N*-(1-phenylprop-2-yn-1-yl)aniline (5)**

Yellow oil, 18.6 mg, 77% yield. ^1^H NMR (400 MHz, CDCl_3_) δ 7.58 – 7.46 (m, 2H), 7.34 – 7.23 (m, 3H), 7.13 – 7.05 (m, 2H), 6.63 – 6.52 (m, 2H), 5.17 (d, *J* = 2.3 Hz, 1H), 2.41 (d, *J* = 2.2 Hz, 1H) ppm. ^13^C NMR (101 MHz, CDCl_3_) δ 144.84, 138.59, 129.07, 128.90, 128.41, 127.20, 123.50, 115.23, 82.55, 73.43, 49.98 ppm.

**3-bromo-*N*-(1-phenylprop-2-yn-1-yl)aniline (6)**

Yellow oil, 20.1 mg, 70% yield. ^1^H NMR (400 MHz, CDCl_3_) δ 7.50 (d, *J* = 7.2 Hz, 2H), 7.37 – 7.24 (m, 3H), 6.97 (t, *J* = 7.9 Hz, 1H), 6.83 (dd, *J* = 8.4, 1.7 Hz, 2H), 6.59 – 6.51 (m, 1H), 5.17 (s, 1H), 4.03 (s, 1H), 2.42 (d, *J* = 2.3 Hz, 1H) ppm. ^13^C NMR (101 MHz, CDCl_3_) δ 147.55, 138.44, 130.50, 128.93, 128.47, 127.23, 123.15, 121.59, 116.72, 112.59, 82.34, 73.55, 49.67 ppm.

**2-((1-phenylprop-2-yn-1-yl)amino)benzaldehyde (7)**

Yellow oil, 15.5 mg, 66% yield. ^1^H NMR (400 MHz, CDCl_3_) δ 9.78 (s, 1H), 8.67 (d, *J* = 6.5 Hz, 1H), 7.49 (ddd, *J* = 21.2, 7.4, 1.7 Hz, 3H), 7.36 – 7.24 (m, 4H), 6.81 – 6.69 (m, 2H), 5.35 (dd, *J* = 6.6, 2.3 Hz, 1H), 2.44 (d, *J* = 2.3 Hz, 1H) ppm. ^13^C NMR (101 MHz, CDCl_3_) δ 194.20, 148.61, 138.11, 136.66, 135.71, 128.99, 128.39, 127.00, 119.28, 116.33, 112.07, 82.04, 73.38, 48.46 ppm.

**4-fluoro-3-methoxy-*N*-(1-phenylprop-2-yn-1-yl)aniline (8)**

Yellow oil, 15.3 mg, 60% yield. ^1^H NMR (400 MHz, CDCl_3_) δ 7.53 (d, *J* = 7.0 Hz, 2H), 7.39 – 7.24 (m, 3H), 6.85 (dd, *J* = 11.2, 8.7 Hz, 1H), 6.30 (dd, *J* = 7.2, 2.7 Hz, 1H), 6.17 (dt, *J* = 8.7, 3.1 Hz, 1H), 5.15 (d, *J* = 2.2 Hz, 1H), 3.75 (s, 3H), 2.43 (d, *J* = 2.2 Hz, 1H) ppm. ^13^C NMR (101 MHz, CDCl_3_) δ 147.96 (d, *J* = 11.6 Hz), 146.48 (d, *J* = 236.3 Hz), 142.98, 138.72, 128.87, 128.40, 127.27, 116.19 (d, *J* = 19.3 Hz), 105.50 (d, *J* = 6.2 Hz), 100.80, 82.73, 73.48, 56.16, 50.60 ppm. ^19^F NMR (377 MHz, Chloroform-*d*) δ -147.75 ppm.

***N*-methyl-*N*-(1-phenylprop-2-yn-1-yl)aniline (9)**^17^

Yellow oil, 16.4 mg, 74% yield. ^1^H NMR (400 MHz, CDCl_3_) δ 7.51 (d, *J* = 7.7 Hz, 2H), 7.33 – 7.20 (m, 5H), 6.92 (d, *J* = 7.9 Hz, 2H), 6.79 (t, *J* = 7.3 Hz, 1H), 5.73 (d, *J* = 2.3 Hz, 1H), 2.64 (s, 3H), 2.45 (d, *J* = 2.3 Hz, 1H) ppm.

***N*-(1-phenylprop-2-yn-1-yl)quinolin-8-amine (10)**

Yellow oil, 16.3 mg, 63% yield. ^1^H NMR (400 MHz, CDCl_3_) δ 8.64 (dd, *J* = 4.2, 1.7 Hz, 1H), 8.00 (dd, *J* = 8.2, 1.7 Hz, 1H), 7.65 – 7.57 (m, 2H), 7.36 – 7.24 (m, 5H), 7.06 (dd, *J* = 8.2, 1.2 Hz, 1H), 6.81 – 6.75 (m, 1H), 6.60 (s, 1H), 5.43 (s, 1H), 2.41 (d, *J* = 2.3 Hz, 1H) ppm. ^13^C NMR (101 MHz, CDCl_3_) δ 147.20, 142.76, 138.96, 138.37, 136.08, 128.86, 128.59, 128.20, 127.48, 127.30, 121.47, 115.46, 106.67, 82.84, 72.93, 49.35 ppm.

***N*-(1-phenylprop-2-yn-1-yl)pyridin-3-amine (11)**

Yellow oil, 9.6 mg, 46% yield. ^1^H NMR (400 MHz, CDCl_3_) δ 8.11 (s, 1H), 7.98 (d, *J* = 4.8 Hz, 1H), 7.53 (dd, *J* = 7.5, 1.8 Hz, 2H), 7.37 – 7.27 (m, 3H), 7.10 (dd, *J* = 8.4, 4.7 Hz, 1H), 7.05 – 6.97 (m, 1H), 5.23 (s, 1H), 4.25 (s, 1H), 2.45 (d, *J* = 2.3 Hz, 1H) ppm. ^13^C NMR (101 MHz, CDCl_3_) δ 147.49, 139.09, 137.97, 136.29, 129.00, 128.61, 127.21, 123.96, 120.79, 81.81, 73.98, 49.53 ppm.

**6-chloro-*N*-(1-phenylprop-2-yn-1-yl)pyridin-3-amine (12)**

Yellow oil, 15.1 mg, 62% yield. ^1^H NMR (400 MHz, CDCl_3_) δ 7.82 (d, *J* = 3.1 Hz, 1H), 7.51 (d, *J* = 6.6 Hz, 2H), 7.38 – 7.25 (m, 3H), 7.06 (d, *J* = 8.6 Hz, 1H), 6.94 (dd, *J* = 8.6, 3.1 Hz, 1H), 5.18 (d, *J* = 2.2 Hz, 1H), 2.45 (d, *J* = 2.3 Hz, 1H) ppm. ^13^C NMR (101 MHz, CDCl_3_) δ 141.38, 140.48, 137.75, 135.94, 129.03, 128.70, 127.21, 124.02, 123.67, 81.57, 74.17, 49.73 ppm.

**5-methyl-*N*-(1-phenylprop-2-yn-1-yl)isoxazol-3-amine (13)**

Yellow solid, 8.9 mg, 42% yield. ^1^H NMR (400 MHz, CDCl_3_) δ 7.52 (d, *J* = 7.3 Hz, 2H), 7.36 – 7.23 (m, 3H), 5.47 (s, 1H), 5.39 (dd, *J* = 8.3, 2.2 Hz, 1H), 4.24 (d, *J* = 8.3 Hz, 1H), 2.45 (d, *J* = 2.3 Hz, 1H), 2.23 (s, 3H) ppm. ^13^C NMR (101 MHz, CDCl_3_) δ 169.22, 163.49, 138.51, 128.77, 128.37, 127.16, 93.48, 82.46, 73.32, 49.51, 12.58 ppm.

***N*-benzyl-1-phenylprop-2-yn-1-amine (14)**^18^

Yellow oil, 15.9 mg, 72% yield. ^1^H NMR (400 MHz, CDCl_3_) δ 7.58 – 7.46 (m, 2H), 7.34 – 7.23 (m, 3H), 7.13 – 7.05 (m, 2H), 6.63 – 6.52 (m, 2H), 5.17 (d, *J* = 2.3 Hz, 1H), 2.41 (d, *J* = 2.2 Hz, 1H) ppm.

**4-(1-phenylprop-2-yn-1-yl)morpholine (15)**^19^

Yellow solid, 18.1 mg, 90% yield. ^1^H NMR (400 MHz, CDCl_3_) δ 7.51 (d, *J* = 7.3 Hz, 2H), 7.31 – 7.20 (m, 3H), 4.52 (d, *J* = 2.4 Hz, 1H), 3.71 – 3.54 (m, 4H), 2.51 (d, *J* = 2.5 Hz, 1H), 2.50 – 2.42 (m, 4H) ppm.

**1-isopropyl-4-(1-phenylprop-2-yn-1-yl)piperazine (16)**

Yellow solid, 22.1 mg, 91% yield. ^1^H NMR (400 MHz, CDCl_3_) δ 7.48 (d, *J* = 7.3 Hz, 2H), 7.30 – 7.24 (m, 2H), 7.24 – 7.20 (m, 1H), 4.51 (d, *J* = 2.3 Hz, 1H), 2.66 – 2.59 (m, 1H), 2.60 – 2.46 (m, 8H), 2.46 (d, *J* = 2.3 Hz, 1H), 0.99 (s, 3H), 0.98 (s, 3H) ppm. ^13^C NMR (101 MHz, CDCl_3_) δ 137.54, 128.45, 128.18, 127.73, 79.72, 75.83, 60.98, 54.53, 48.67, 18.56, 18.51 ppm.

**1-cyclopropyl-4-(1-phenylprop-2-yn-1-yl)piperazine (17)**

Yellow solid, 21.2 mg, 88% yield. ^1^H NMR (400 MHz, CDCl_3_) δ 7.49 (d, *J* = 7.4 Hz, 2H), 7.30 – 7.24 (m, 2H), 7.24 – 7.17 (m, 1H), 4.53 (d, *J* = 2.3 Hz, 1H), 2.58 (s, 4H), 2.48 (d, *J* = 15.4 Hz, 4H), 2.44 (d, *J* = 2.3 Hz, 1H), 1.56 – 1.48 (m, 1H), 0.41 – 0.27 (m, 4H) ppm. ^13^C NMR (101 MHz, CDCl_3_) δ 137.59, 128.43, 128.18, 127.71, 79.72, 75.84, 61.00, 53.40, 38.45, 5.70, 5.67 ppm.

**1-(oxetan-3-yl)-4-(1-phenylprop-2-yn-1-yl)piperazine (18)**

White solid, 22.8 mg, 89% yield. ^1^H NMR (400 MHz, CDCl_3_) δ δ 7.58 (d, *J* = 7.5 Hz, 2H), 7.37 (dd, *J* = 8.2, 6.4 Hz, 2H), 7.34 – 7.29 (m, 1H), 4.71 – 4.59 (m, 5H), 3.58 – 3.46 (m, 1H), 2.72 – 2.60 (m, 4H), 2.58 (d, *J* = 2.3 Hz, 1H), 2.48 – 2.28 (m, 4H) ppm. ^13^C NMR (101 MHz, CDCl_3_) δ 137.32, 128.39, 128.22, 127.81, 79.30, 76.08, 75.46, 60.94, 59.21, 49.66 ppm.

**cyclopropyl(4-(1-phenylprop-2-yn-1-yl)piperazin-1-yl)methanone (19)**

Yellow oil, 22.5 mg, 84% yield. ^1^H NMR (400 MHz, CDCl_3_) δ 7.51 (d, *J* = 7.4 Hz, 2H), 7.33 – 7.21 (m, 3H), 4.60 (d, *J* = 2.3 Hz, 1H), 3.71 – 3.48 (m, 4H), 2.50 (d, *J* = 2.3 Hz, 1H), 2.52 – 2.44 (m, 4H), 1.68 – 1.60 (m, 1H), 0.94 – 0.88 (m, 2H), 0.71 – 0.63 (m, 2H). ppm. ^13^C NMR (101 MHz, CDCl_3_) δ 171.92, 137.07, 128.37, 128.30, 127.96, 78.79, 76.38, 60.98, 49.38, 49.02, 45.52, 42.15, 10.93, 7.38 ppm.

**1-(1-phenylprop-2-yn-1-yl)-1,2,3,4-tetrahydroquinoline (20)**

Yellow oil, 17.8 mg, 72% yield. ^1^H NMR (400 MHz, CDCl_3_) δ 7.51 (d, *J* = 8.2 Hz, 2H), 7.34 – 7.26 (m, 2H), 7.26 – 7.20 (m, 1H), 7.05 – 6.99 (m, 1H), 6.95 (d, *J* = 8.8 Hz, 1H), 6.80 (d, *J* = 8.3 Hz, 1H), 6.62 (t, *J* = 7.3 Hz, 1H), 5.80 (d, *J* = 2.4 Hz, 1H), 3.12 (dt, *J* = 11.9, 6.3 Hz, 1H), 2.93 – 2.84 (m, 1H), 2.80 – 2.61 (m, 2H), 2.41 (d, *J* = 2.4 Hz, 1H), 1.87 – 1.78 (m, 2H) ppm. ^13^C NMR (101 MHz, CDCl_3_) δ 145.14, 137.64, 129.32, 128.50, 127.74, 127.47, 126.99, 124.65, 117.52, 111.96, 80.42, 74.20, 53.69, 44.06, 28.07, 22.32 ppm.

**1-(1-phenylprop-2-yn-1-yl)-4-(4-(trifluoromethyl)phenoxy)piperidine (21)**

Yellow oil, 32.3 mg, 90% yield. ^1^H NMR (400 MHz, CDCl_3_) δ 7.52 (d, *J* = 7.1 Hz, 2H), 7.34 – 7.20 (m, 3H), 7.04 (d, *J* = 8.7 Hz, 2H), 6.80 (d, *J* = 9.1 Hz, 2H), 4.61 (s, 1H), 4.28 – 4.10 (m, 1H), 2.84 – 2.69 (m, 1H), 2.68 – 2.54 (m, 1H), 2.50 (d, *J* = 2.3 Hz, 1H), 2.50 – 2.42 (m, 1H), 2.31 (t, *J* = 10.2 Hz, 1H), 2.00 – 1.83 (m, 2H), 1.84 – 1.60 (m, 2H) ppm. ^13^C NMR (101 MHz, CDCl_3_) δ 156.02, 142.69 (q, *J* = 2.0 Hz), 137.67, 128.28, 128.22, 127.78, 122.43, 120.58 (q, *J* = 256.0 Hz), 116.85, 79.33, 75.92, 73.57, 61.01, 47.75, 45.65, 31.06, 30.87 ppm. ^19^F NMR (377 MHz, Chloroform-*d*) δ -58.37 ppm.

***N*-(1-phenylprop-2-yn-1-yl)cyclohexanamine (22)**

Yellow oil, 14.3 mg, 67% yield. ^1^H NMR (400 MHz, CDCl_3_) δ 7.46 (d, *J* = 7.0 Hz, 2H), 7.31 – 7.25 (m, 2H), 7.24 – 7.20 (m, 1H), 4.61 (d, *J* = 2.2 Hz, 1H), 2.77 – 2.65 (m, 1H), 2.40 (d, *J* = 2.2 Hz, 1H), 1.93 – 1.86 (m, 1H), 1.73 – 1.54 (m, 5H), 1.24 – 1.07 (m, 5H) ppm. ^13^C NMR (101 MHz, CDCl_3_) δ 140.63, 128.59, 127.73, 127.42, 84.46, 72.85, 54.19, 50.79, 33.97, 32.59, 26.16, 25.00, 24.73 ppm.

***N*-(1-phenylprop-2-yn-1-yl)cyclopentanamine (23)**

Yellow oil, 8.1 mg, 41% yield. ^1^H NMR (400 MHz, CDCl_3_) δ 7.45 (d, *J* = 7.3 Hz, 2H), 7.28 (dd, *J* = 8.2, 6.5 Hz, 2H), 7.24 – 7.21 (m, 1H), 4.49 (d, *J* = 2.2 Hz, 1H), 3.37 – 3.26 (m, 1H), 2.40 (d, *J* = 2.2 Hz, 1H), 1.75 – 1.39 (m, 8H) ppm. ^13^C NMR (101 MHz, CDCl_3_) δ 140.16, 128.58, 127.83, 127.52, 84.17, 73.06, 57.30, 52.71, 33.34, 32.59, 24.08, 24.07 ppm.

**3-(1-Phenylprop-2-ynyl)-1H-indole (25)**^17^

Yellow oil, 12.9 mg, 56% yield. ^1^H NMR (400 MHz, CDCl_3_) δ 8.02 (br s, 1H), 7.46 – 7.35 (m, 3H), 7.30 – 7.21 (m, 3H), 7.17 – 7.07 (m, 2H), 7.05 (dd, *J* = 2.6, 1.0 Hz, 1H), 6.98 (t, *J* = 7.0 Hz, 1H), 5.19 (d, *J* = 2.6 Hz, 1H), 2.36 (d, *J* = 2.6 Hz, 1H) ppm.

**2-chloro-5-((1-phenylprop-2-yn-1-yl)oxy)pyridine (27)**

Yellow oil, 14.1 mg, 58% yield. ^1^H NMR (400 MHz, CDCl_3_) δ 8.15 (d, *J* = 3.0 Hz, 1H), 7.56 – 7.49 (m, 2H), 7.39 – 7.33 (m, 3H), 7.30 (dd, *J* = 8.7, 3.1 Hz, 1H), 7.20 – 7.18 (m, 1H), 5.76 (d, *J* = 2.2 Hz, 1H), 2.70 (d, *J* = 2.2 Hz, 1H) ppm. ^13^C NMR (101 MHz, CDCl_3_) δ 152.59, 143.72, 138.68, 136.16, 129.39, 128.91, 127.40, 126.87, 124.39, 79.53, 78.08, 70.87 ppm.

**2-methyl-1-phenylnaphtho[2,1-b]furan (29)**^20^

Yellow oil, 10.9 mg, 42% yield. ^1^H NMR (400 MHz, CDCl_3_) δ 7.84 (d, *J* = 6.8 Hz, 1H), 7.69 (d, *J* = 8.4 Hz, 1H), 7.63 – 7.54 (m, 2H), 7.48 – 7.38 (m, 5H), 7.34 – 7.27 (m, 1H), 7.24 – 7.19 (m, 1H), 2.36 (s, 3H) ppm.

**5-methylene-3-(2-morpholinoethyl)-4-phenyloxazolidin-2-one (31)**

Yellow oil, 17.3 mg, 60% yield. ^1^H NMR (400 MHz, CDCl_3_) δ 7.38 – 7.29 (m, 3H), 7.25 – 7.19 (m, 4H), 5.39 (t, *J* = 2.4 Hz, 1H), 4.69 (t, *J* = 2.9 Hz, 1H), 3.98 (dd, *J* = 3.2, 2.2 Hz, 1H), 3.67 – 3.48 (m, 5H), 2.90 – 2.76 (m, 1H), 2.45 – 2.21 (m, 6H) ppm. ^13^C NMR (101 MHz, CDCl_3_) δ 155.29, 154.97, 137.28, 129.28, 127.56, 88.02, 66.87, 63.48, 55.91, 53.50, 38.11 ppm.

***N*-(4-ethylhex-1-yn-3-yl)aniline (33)**^17^

Colorless oil, 14.1 mg, 70% yield. ^1^H NMR (400 MHz, CDCl_3_) δ 7.24 – 7.14 (m, 2H), 6.76 (t, *J* = 7.3 Hz, 1H), 6.70 (d, *J* = 7.4 Hz, 2H), 4.19 (s, 1H), 3.74 (s, 1H), 2.20 (d, *J* = 2.2 Hz, 1H), 1.69 – 1.45 (m, 5H), 1.01 – 0.95 (m, 6H) ppm.

***N*-(3,4-dimethylpent-1-yn-3-yl)aniline (35)**

Yellow oil, 13.5 mg, 72% yield. ^1^H NMR (400 MHz, CDCl_3_) δ 7.13 – 7.03 (m, 2H), 6.91 – 6.85 (m, 2H), 6.70 (t, *J* = 7.3 Hz, 1H), 3.89 (d, *J* = 6.0 Hz, 1H), 2.33 (s, 1H), 2.11 – 2.00 (m, 1H), 1.40 (s, 3H), 1.07 (d, *J* = 6.8 Hz, 3H), 0.94 (d, *J* = 6.8 Hz, 3H) ppm. ^13^C NMR (101 MHz, CDCl_3_) δ 145.65, 128.81, 118.52, 116.60, 86.43, 72.32, 55.44, 36.58, 23.84, 17.96, 16.81 ppm.

***N*-(3-methyl-5-phenylpent-1-yn-3-yl)aniline (37)**^21^

Yellow oil, 19.5 mg, 78% yield. ^1^H NMR (400 MHz, CDCl_3_) δ 7.32 (t, *J* = 7.5 Hz, 2H), 7.27 – 7.13 (m, 5H), 6.97 (d, *J* = 8.0 Hz, 2H), 6.83 (t, *J* = 7.3 Hz, 1H), 3.68 (s, 1H), 3.01 – 2.79 (m, 2H), 2.50 (s, 1H), 2.27 – 2.03 (m, 2H) ppm.

**2-phenyl-2-(phenylamino)but-3-yn-1-ol (39)**^22^

Yellow oil, 14.9 mg, 63% yield. ^1^H NMR (400 MHz, CDCl_3_) δ 7.65 – 7.59 (m, 2H), 7.33 – 7.24 (m, 3H), 7.05 – 6.96 (m, 2H), 6.66 (t, *J* = 7.4 Hz, 1H), 6.49 (d, *J* = 7.6 Hz, 2H), 3.70 (d, *J* = 10.9 Hz, 1H), 3.56 (d, *J* = 10.9 Hz, 1H), 2.47 (s, 1H) ppm.

***N*-methyl-2-phenyl-*N*-(3-phenyl-3-(4-(trifluoromethyl)phenoxy)propyl)but-3-yn-1-amine (40)**

Colorless oil, 34.3 mg, 81% yield. ^1^H NMR (400 MHz, CDCl_3_) δ 7.44 (d, *J* = 5.7 Hz, 1H), 7.40 – 7.34 (m, 2H), 7.32 (d, *J* = 8.7 Hz, 1H), 7.24 (d, *J* = 4.3 Hz, 2H), 7.23 – 7.02 (m, 6H), 6.82 (d, *J* = 8.6 Hz, 1H), 6.70 (d, *J* = 8.6 Hz, 1H), 5.21 (ddd, *J* = 12.5, 8.5, 4.5 Hz, 1H), 4.62 (dd, *J* = 15.8, 2.3 Hz, 1H), 2.71 – 2.46 (m, 2H), 2.44 (dd, *J* = 7.8, 2.3 Hz, 1H), 2.14 (d, *J* = 35.4 Hz, 3H), 2.09 – 2.01 (m, 1H), 1.97 – 1.82 (m, 1H), 1.52 (s, 1H) ppm. ^13^C NMR (101 MHz, CDCl_3_) δ 160.77 (d, *J* = 25.2 Hz), 141.42 (d, *J* = 17.1 Hz), 138.14 (d, *J* = 19.0 Hz), 128.75 (d, *J* = 6.5 Hz), 128.26 (d, *J* = 20.2 Hz), 128.08 (d, *J* = 5.4 Hz), 127.73 (d, *J* = 2.0 Hz), 127.62 (d, *J* = 12.0 Hz), 126.76 (d, *J* = 3.7 Hz), 126.60 (q, *J* = 3.8 Hz), 125.81 (d, *J* = 7.7 Hz), 122.62 (q, *J* = 15.0 Hz), 115.74 (d, *J* = 1.5 Hz), 78.85 (d, *J* = 12.7 Hz), 77.95 (d, *J* = 41.4 Hz), 75.97 (d, *J* = 6.8 Hz), 60.54 (d, *J* = 21.4 Hz), 50.02 (d, *J* = 129.5 Hz), 37.86 (d, *J* = 109.0 Hz), 36.92 (d, *J* = 17.8 Hz), 27.77 ppm. ^19^F NMR (377 MHz, Chloroform-*d*) δ -61.47 ppm.

***N*-(3-(9,10-ethanoanthracen-9(10H)-yl)propyl)-*N*-methyl-1-phenylprop-2-yn-1-amine (41)**

Yellow oil, 36.1 mg, 92% yield. ^1^H NMR (400 MHz, CDCl_3_) δ 7.63 – 7.56 (m, 2H), 7.33 – 7.26 (m, 2H), 7.26 – 7.20 (m, 1H), 7.19 – 7.11 (m, 4H), 7.05 – 6.96 (m, 4H), 4.79 (d, *J* = 2.3 Hz, 1H), 4.18 (t, *J* = 2.8 Hz, 1H), 2.75 – 2.59 (m, 2H), 2.52 (d, *J* = 2.3 Hz, 1H), 2.46 – 2.25 (m, 2H), 2.24 (s, 3H), 1.96 – 1.79 (m, 2H), 1.78 – 1.68 (m, 2H), 1.52 – 1.44 (m, 2H) ppm. ^13^C NMR (101 MHz, CDCl_3_) δ 145.58, 145.06, 128.44, 128.24, 127.72, 125.29, 125.20, 123.33, 121.41, 121.39, 79.14, 75.97, 60.35, 55.16, 44.85, 44.59, 38.32, 29.68, 28.82, 27.72, 23.19 ppm.

***N*-methyl-*N*-(naphthalen-2-ylmethyl)-1-phenylprop-2-yn-1-amine (42)**

Yellow oil, 22.8 mg, 80% yield. ^1^H NMR (400 MHz, CDCl_3_) δ 8.17 (d, *J* = 7.3 Hz, 1H), 7.79 – 7.74 (m, 1H), 7.70 (d, *J* = 8.2 Hz, 1H), 7.49 – 7.38 (m, 5H), 7.33 (dd, *J* = 8.2, 6.9 Hz, 1H), 7.26 – 7.17 (m, 3H), 4.65 (s, 1H), 4.09 (d, *J* = 12.9 Hz, 1H), 3.91 (d, *J* = 12.9 Hz, 1H), 2.59 (d, *J* = 2.3 Hz, 1H), 2.12 (s, 3H) ppm. ^13^C NMR (101 MHz, CDCl_3_) δ 138.40, 134.39, 133.97, 132.63, 128.43, 128.34, 128.21, 128.09, 127.81, 127.56, 125.76, 125.67, 125.19, 124.95, 78.57, 76.45, 58.71, 57.43, 37.54 ppm.

**(2*S*,6*R*)-2,6-dimethyl-4-(1-phenylprop-2-yn-1-yl)morpholine (43)**

Colorless oil, 21.6 mg, 94% yield. ^1^H NMR (400 MHz, CDCl_3_) δ 7.50 (d, *J* = 7.2 Hz, 2H), 7.31 – 7.20 (m, 3H), 4.53 (d, *J* = 2.3 Hz, 1H), 3.73 – 3.65 (m, 1H), 3.58 – 3.41 (m, 1H), 2.64 (d, *J* = 11.0 Hz, 1H), 2.49 (d, *J* = 2.3 Hz, 1H), 2.32 (d, *J* = 11.1 Hz, 1H), 2.20 (t, *J* = 10.6 Hz, 1H), 1.96 – 1.80 (m, 1H), 1.12 (d, *J* = 6.3 Hz, 3H), 1.02 (d, *J* = 6.3 Hz, 3H) ppm. ^13^C NMR (101 MHz, CDCl_3_) δ 137.17, 128.45, 128.25, 127.84, 79.15, 76.18, 71.75, 60.96, 58.04, 52.65, 19.14 ppm.

**5-(1-phenylprop-2-yn-1-yl)-4,5,6,7-tetrahydrothieno[3,2-c]pyridine (44)**

Yellow oil, 22.8 mg, 90% yield. ^1^H NMR (400 MHz, CDCl_3_) δ 7.59 (d, *J* = 7.5 Hz, 2H), 7.35 – 7.21 (m, 3H), 6.99 (d, *J* = 5.1 Hz, 1H), 6.62 (d, *J* = 5.2 Hz, 1H), 4.83 (d, *J* = 2.3 Hz, 1H), 3.63 (q, *J* = 14.4 Hz, 2H), 2.92 – 2.73 (m, 4H), 2.52 (d, *J* = 2.3 Hz, 1H) ppm. ^13^C NMR (101 MHz, CDCl_3_) δ 137.14, 133.58, 133.11, 128.47, 128.37, 128.04, 125.31, 122.91, 78.95, 76.44, 60.66, 49.18, 47.35, 25.68 ppm.

**5,6-dimethoxy-2-((1-(1-phenylprop-2-yn-1-yl)piperidin-4-yl)methyl)-2,3-dihydro-1H-inden-1-one (45)**

Yellow oil, 35.5 mg, 88% yield. ^1^H NMR (400 MHz, CDCl_3_) δ 7.52 (d, *J* = 7.1 Hz, 2H), 7.32 – 7.25 (m, 2H), 7.25 – 7.20 (m, 1H), 7.09 (s, 1H), 6.77 (s, 1H), 4.63 (s, 1H), 3.88 (s, 3H), 3.83 (s, 3H), 3.15 (dd, *J* = 17.6, 8.1 Hz, 1H), 2.92 (d, *J* = 11.1 Hz, 1H), 2.61 (dq, *J* = 13.3, 5.2, 4.5 Hz, 3H), 2.50 (d, *J* = 1.5 Hz, 1H), 2.45 (t, *J* = 10.4 Hz, 1H), 2.12 (t, *J* = 12.8 Hz, 1H), 1.86 – 1.52 (m, 3H), 1.51 – 1.33 (m, 2H), 1.30 – 1.16 (m, 2H) ppm. ^13^C NMR (101 MHz, CDCl_3_) δ 207.78, 155.50, 149.46, 148.77, 129.29, 128.52, 128.20, 128.19, 127.85, 107.38, 104.40, 79.44, 75.97, 61.29, 56.22, 56.10, 52.35, 47.10, 45.42, 45.40, 38.59, 34.21, 33.37, 33.33 ppm.

**8-chloro-11-(1-(1-phenylprop-2-yn-1-yl)piperidin-4-ylidene)-6,11-dihydro-5H-benzo[5,6]cyclohepta[1,2-b]pyridine (46)**

Yellow oil, 23.4 mg, 55% yield. ^1^H NMR (400 MHz, CDCl_3_) δ 8.30 (ddd, *J* = 10.8, 4.8, 1.7 Hz, 1H), 7.50 (d, *J* = 7.2 Hz, 2H), 7.35 (t, *J* = 6.7 Hz, 1H), 7.26 (t, *J* = 7.3 Hz, 2H), 7.22 – 7.19 (m, 1H), 7.10 – 6.95 (m, 4H), 4.57 (s, 1H), 3.39 – 3.24 (m, 2H), 2.80 – 2.65 (m, 3H), 2.58 – 2.48 (m, 1H), 2.44 (d, *J* = 2.3 Hz, 1H), 2.43 – 2.11 (m, 6H) ppm. ^13^C NMR (101 MHz, CDCl_3_) δ 157.72, 146.61, 146.59, 139.54, 139.50, 138.91, 137.81, 137.22, 137.19, 133.47, 133.44, 132.70, 132.63, 132.61, 130.92, 128.98, 128.95, 128.29, 128.27, 128.14, 127.68, 125.99, 125.96, 122.08, 79.54, 75.68, 60.98, 60.96, 52.28, 52.22, 49.37, 31.88, 31.44, 31.22, 31.01, 30.77 ppm.

**1-(1-phenylprop-2-yn-1-yl)-4-(5-(trifluoromethyl)pyridin-2-yl)piperazine (47)**

White solid, 25.2 mg, 73% yield. ^1^H NMR (400 MHz, CDCl_3_) δ 8.31 (s, 1H), 7.53 (dd, *J* = 8.3, 3.6 Hz, 3H), 7.35 – 7.21 (m, 3H), 6.53 (d, *J* = 9.0 Hz, 1H), 4.63 (d, *J* = 2.3 Hz, 1H), 3.58 (t, *J* = 5.1 Hz, 4H), 2.57 (t, *J* = 5.2 Hz, 4H), 2.49 (d, *J* = 2.3 Hz, 1H) ppm. ^13^C NMR (101 MHz, CDCl_3_) δ 160.34, 145.75 (d, *J* = 4.3 Hz), 137.18, 134.43 (q, *J* = 3.3 Hz), 128.40, 128.32, 127.96, 124.64 (d, *J* = 270.4 Hz), 115.02 (d, *J* = 32.8 Hz), 105.50, 78.86, 76.36, 61.05, 48.87, 44.74 ppm. ^19^F NMR (377 MHz, Chloroform-*d*) δ -61.11 ppm.

**1-benzhydryl-4-(1-phenylprop-2-yn-1-yl)piperazine (48)**

Yellow solid, 26.8 mg, 73% yield. ^1^H NMR (400 MHz, CDCl_3_) δ 7.46 (d, *J* = 7.1 Hz, 2H), 7.33 (t, *J* = 9.5 Hz, 4H), 7.24 (dd, *J* = 8.2, 6.3 Hz, 2H), 7.21 – 7.12 (m, 5H), 7.08 (dt, *J* = 10.2, 7.4 Hz, 2H), 4.53 (d, *J* = 2.3 Hz, 1H), 4.15 (s, 1H), 2.55 – 2.46 (m, 4H), 2.49 (d, *J* = 2.4 Hz, 1H), 2.42 – 2.24 (m, 4H) ppm. ^13^C NMR (101 MHz, CDCl_3_) δ 142.79, 137.47, 128.44, 128.17, 128.03, 128.00, 127.74, 126.91, 79.77, 76.21, 75.80, 60.90, 51.96, 49.37 ppm.

**1-(bis(4-fluorophenyl)methyl)-4-(1-phenylprop-2-yn-1-yl)piperazine (49)**

Yellow solid, 31.4 mg, 78% yield. ^1^H NMR (400 MHz, CDCl_3_) δ 7.46 (d, *J* = 7.2 Hz, 2H), 7.28 – 7.17 (m, 7H), 6.91 – 6.81 (m, 4H), 4.53 (d, *J* = 2.4 Hz, 1H), 4.13 (s, 1H), 2.57 – 2.42 (m, 5H), 2.37 – 2.18 (m, 4H) ppm. ^13^C NMR (101 MHz, CDCl_3_) δ 161.80 (d, *J* = 244.8 Hz), 138.26, 137.36, 129.31 (dd, *J* = 7.8, 2.6 Hz), 128.43, 128.18, 127.78, 115.36 (dd, *J* = 21.2, 4.2 Hz), 79.61, 75.87, 74.46, 60.86, 51.78, 49.19 ppm. ^19^F NMR (377 MHz, CDCl_3_) δ -115.78 ppm.

**furan-2-yl(4-(1-phenylprop-2-yn-1-yl)piperazin-1-yl)methanone (50)**

Yellow soild, 25.0 mg, 85% yield. ^1^H NMR (400 MHz, CDCl_3_) δ 7.55 – 7.48 (m, 2H), 7.38 (dd, *J* = 1.8, 0.8 Hz, 1H), 7.32 – 7.21 (m, 3H), 6.90 (dd, *J* = 3.5, 0.9 Hz, 1H), 6.39 (dd, *J* = 3.4, 1.8 Hz, 1H), 4.61 (d, *J* = 2.3 Hz, 1H), 3.73 (br, 4H), 2.56 – 2.50 (m, 4H), 2.50 (d, *J* = 2.3 Hz, 1H) ppm. ^13^C NMR (101 MHz, CDCl_3_) δ 159.04, 147.96, 143.62, 136.98, 128.38, 128.32, 128.00, 116.32, 111.23, 78.71, 76.48, 60.97, 49.31 ppm.

**7-chloro-4-(4-(1-phenylprop-2-yn-1-yl)piperazin-1-yl)quinoline (51)**

Yellow solid, 31.5 mg, 87% yield. ^1^H NMR (400 MHz, CDCl_3_) δ 8.62 (d, *J* = 5.0 Hz, 1H), 7.96 (d, *J* = 2.1 Hz, 1H), 7.88 (d, *J* = 9.0 Hz, 1H), 7.55 (d, *J* = 7.4 Hz, 2H), 7.35 – 7.28 (m, 3H), 7.27 – 7.22 (m, 1H), 6.73 (d, *J* = 5.0 Hz, 1H), 4.68 (d, *J* = 2.3 Hz, 1H), 3.23 – 3.08 (m, 4H), 2.76 (t, *J* = 4.9 Hz, 4H), 2.58 (d, *J* = 2.3 Hz, 1H) ppm. ^13^C NMR (101 MHz, CDCl_3_) δ 157.14, 151.73, 149.89, 137.19, 135.00, 128.61, 128.40, 128.33, 127.98, 126.14, 125.36, 121.87, 108.90, 79.06, 76.42, 60.98, 52.26, 49.07 ppm.

**4-((1-phenylprop-2-yn-1-yl)amino)benzoic acid**

Yellow solid, 10.1 mg, 40% yield. ^1^H NMR (400 MHz, CDCl_3_) δ 7.89 (d, *J* = 8.7 Hz, 2H), 7.52 (d, *J* = 7.2 Hz, 2H), 7.40 – 7.27 (m, 3H), 6.65 (d, *J* = 8.7 Hz, 2H), 5.31 (s, 1H), 4.49 (s, 1H), 2.46 (d, *J* = 2.3 Hz, 1H) ppm. ^13^C NMR (101 MHz, CDCl_3_) δ 171.35, 150.56, 138.06, 132.21, 129.02, 128.61, 127.20, 118.81, 112.72, 81.91, 73.70, 49.26 ppm.

1. **Supplementary references**

[1] J. Song, C. Chen, S. Zhu, M. Zhu, J. Dai, U. Ray, Y. Li, Y. Kuang, Y. Li, N. Quispe, Y. Yao, A. Gong, U. H. Leiste, H. A. Bruck, J. Y. Zhu, A. Vellore, H. Li, M. L. Minus, Z. Jia, A. Martini, T. Li, L. Hu, *Nature* **2018**, *554*, 224.

[2] F. Chen, A. S. Gong, M. Zhu, G. Chen, S. D. Lacey, F. Jiang, Y. Li, Y. Wang, J. Dai, Y. Yao, J. Song, B. Liu, K. Fu, S. Das, L. Hu, *ACS Nano* **2017**, *11*, 4275.

[3] C. Chen, Y. Zhang, Y. Li, J. Dai, J. Song, Y. Yao, Y. Gong, I. Kierzewski, J. Xie, L. Hu, *Energy Environ. Sci*. **2017**, *10*, 538.

[4] X. Zhang, X. Xie, H. Wang, J. Zhang, B. Pan, Y. Xie, *J. Am. Chem. Soc.* **2013**, *135*, 18.

[5] Y. Zhao, R. Wei, X. Feng, L. Sun, P. Liu, Y. Su, L. Shi, *ACS Appl. Mater. Interfaces* **2016**, *8*, 21555.

[6] J. Liu, W. Fang, Z. Wei, Z. Qin, Z. Jiang, W. Shangguan, *Appl. Catal. B Environ*. **2018**, *238*, 465.

[7] T. Sano, H. Sato, T. Hori, T. Hirakawa, Y. Teramoto, K. Koike, *Mol. Catal*. **2019**, *474*, 110451.

[8] G. Kresse, J. Hafner, *Phys. Rev. B* **1993**, *47*, 558.

[9] J. P. Perdew, K. Burke, M. Ernzerhof, *Phys. Rev. Lett.* **1996**, *77*, 3865

[10] A. Tkatchenko, M. Scheffler, *Phys. Rev. Lett.* **2009**, *102*, 073005.

[11] S. Grimme, J. Antony, S. Ehrlich, H. Krieg, *J. Chem. Phys.* **2010**, *132*, 154104.

[12] M. J. Frisch, G. W. Trucks, H. B. Schlegel, Gaussian 16, **2016**.

[13] K. Wang, Y. Li, J. Li, G. Zhang, *Appl. Catal. B Environ*. **2020**, *263*, 117730.

[14] L. Cao, Q. Luo, W. Liu, Y. Lin, X. Liu, Y. Cao, W. Zhang, Y. Wu, J. Yang, T. Yao, S. Wei, *Nat. Catal*. **2019**, *2*, 134.

[15] S. Wei, A. Li, J.-C. Liu, Z. Li, W. Chen, Y. Gong, Q. Zhang, W.-C. Cheong, Y. Wang, L. Zheng, H. Xiao, C. Chen, D. Wang, Q. Peng, L. Gu, X. Han, J. Li, Y. Li, *Nat. Nanotechnol*. **2018**, *13*, 856.

[16] C. Zhao, X. Dai, T. Yao, W. Chen, X. Wang, J. Wang, J. Yang, S. Wei, Y. Wu, Y. Li, *J. Am. Chem. Soc*. **2017**, *139*, 8078.

[17] Q. Cai, H. Rao, S.-J. Li, Y. Lan, K. Ding, X. Wang, *Chem* **2024**, *10*, 265.

[18] D. D. Roberts, M. G. McLaughlin, *Org. Lett.* **2021**, *23*, 4463.

[19] T. Sugiishi, A. Kimura, H. Nakamura, *J. Am. Chem. Soc.* **2010**, *132*, 5332.

[20] L. Shao, Y.-H. Wang, D.-Y. Zhang, J. Xu, X.-P. Hu, *Angew. Chem. Int. Ed.* **2016**, *55*, 5014.

[21] Z. Zhang, Y. Sun, Y. Gong, D.-L. Tang, H. Luo, Z.-P. Zhao, F. Zhou, X. Wang, J. Zhou, *Nat. Chem.* **2024**, *16*, 521.

[22] L. Tian, L. Gong, X. Zhang, *Adv. Synth. Catal.* **2018**, *360*, 2055.

1. **Copies of NMR Spectra**

**^1^H NMR spectra (400 MHz, CDCl_3_) of 3**

**^1^H NMR spectra (400 MHz, CDCl_3_) of 4**

**^1^H NMR spectra (400 MHz, CDCl_3_) of 5**

**^13^C NMR spectra (101 MHz, CDCl_3_) of 5**

**^1^H NMR spectra (400 MHz, CDCl_3_) of 6**

**^13^C NMR spectra (101 MHz, CDCl_3_) of 6**

**^1^H NMR spectra (400 MHz, CDCl_3_) of 7**

**^13^C NMR spectra (101 MHz, CDCl_3_) of 7**

**^1^H NMR spectra (400 MHz, CDCl_3_) of 8**

**^13^C NMR spectra (101 MHz, CDCl_3_) of 8**

**^19^F NMR spectra (376 MHz, CDCl_3_) of 8**

**^1^H NMR spectra (400 MHz, CDCl_3_) of 9**

**^1^H NMR spectra (400 MHz, CDCl_3_) of 10**

**^13^C NMR spectra (101 MHz, CDCl_3_) of 10**

**^1^H NMR spectra (400 MHz, CDCl_3_) of 11**

**^13^C NMR spectra (101 MHz, CDCl_3_) of 11**

**^1^H NMR spectra (400 MHz, CDCl_3_) of 12**

**^13^C NMR spectra (101 MHz, CDCl_3_) of 12**

**^1^H NMR spectra (400 MHz, CDCl_3_) of 13**

**^13^C NMR spectra (101 MHz, CDCl_3_) of 13**

**^1^H NMR spectra (400 MHz, CDCl_3_) of 14**

**^1^H NMR spectra (400 MHz, CDCl_3_) of 15**

**^1^H NMR spectra (400 MHz, CDCl_3_) of 16**

**^13^C NMR spectra (101 MHz, CDCl_3_) of 16**

**^1^H NMR spectra (400 MHz, CDCl_3_) of 17**

**^13^C NMR spectra (101 MHz, CDCl_3_) of 17**

**^1^H NMR spectra (400 MHz, CDCl_3_) of 18**

**^13^C NMR spectra (101 MHz, CDCl_3_) of 18**

**^1^H NMR spectra (400 MHz, CDCl_3_) of 19**

**^13^C NMR spectra (101 MHz, CDCl_3_) of 19**

**^1^H NMR spectra (400 MHz, CDCl_3_) of 20**

**^13^C NMR spectra (101 MHz, CDCl_3_) of 20**

**^1^H NMR spectra (400 MHz, CDCl_3_) of 21**

**^13^C NMR spectra (101 MHz, CDCl_3_) of 21**

**^19^F NMR spectra (376 MHz, CDCl_3_) of 21**

**^1^H NMR spectra (400 MHz, CDCl_3_) of 22**

**^13^C NMR spectra (101 MHz, CDCl_3_) of 22**

**^1^H NMR spectra (400 MHz, CDCl_3_) of 23**

**^13^C NMR spectra (101 MHz, CDCl_3_) of 23**

**^1^H NMR spectra (400 MHz, CDCl_3_) of 25**

**^1^H NMR spectra (400 MHz, CDCl_3_) of 27**

**^13^C NMR spectra (101 MHz, CDCl_3_) of 27**

**^1^H NMR spectra (400 MHz, CDCl_3_) of 29**

**^1^H NMR spectra (400 MHz, CDCl_3_) of 31**

**^13^C NMR spectra (101 MHz, CDCl_3_) of 31**

**^1^H NMR spectra (400 MHz, CDCl_3_) of 33**

**^1^H NMR spectra (400 MHz, CDCl_3_) of 35**

**^13^C NMR spectra (101 MHz, CDCl_3_) of 35**

**^1^H NMR spectra (400 MHz, CDCl_3_) of 37**

**^1^H NMR spectra (400 MHz, CDCl_3_) of 39**

**^1^H NMR spectra (400 MHz, CDCl_3_) of 40**

**^13^C NMR spectra (101 MHz, CDCl_3_) of 40**

**^19^F NMR spectra (376 MHz, CDCl_3_) of 40**

**^1^H NMR spectra (400 MHz, CDCl_3_) of 41**

**^13^C NMR spectra (101 MHz, CDCl_3_) of 41**

**^1^H NMR spectra (400 MHz, CDCl_3_) of 42**

**^13^C NMR spectra (101 MHz, CDCl_3_) of 42**

**^1^H NMR spectra (400 MHz, CDCl_3_) of 43**

**^13^C NMR spectra (101 MHz, CDCl_3_) of 43**

**^1^H NMR spectra (400 MHz, CDCl_3_) of 44**

**^13^C NMR spectra (101 MHz, CDCl_3_) of 44**

**^1^H NMR spectra (400 MHz, CDCl_3_) of 45**

**^13^C NMR spectra (101 MHz, CDCl_3_) of 45**

**^1^H NMR spectra (400 MHz, CDCl_3_) of 46**

**^13^C NMR spectra (101 MHz, CDCl_3_) of 46**

**^1^H NMR spectra (400 MHz, CDCl_3_) of 47**

**^13^C NMR spectra (101 MHz, CDCl_3_) of 47**

**^19^F NMR spectra (376 MHz, CDCl_3_) of 47**

**^1^H NMR spectra (400 MHz, CDCl_3_) of 48**

**^13^C NMR spectra (101 MHz, CDCl_3_) of 48**

**^1^H NMR spectra (400 MHz, CDCl_3_) of 49**

**^13^C NMR spectra (101 MHz, CDCl_3_) of 49**

**^19^F NMR spectra (376 MHz, CDCl_3_) of 49**

**^1^H NMR spectra (400 MHz, CDCl_3_) of 50**

**^13^C NMR spectra (101 MHz, CDCl_3_) of 50**

**^1^H NMR spectra (400 MHz, CDCl_3_) of 51**

**^13^C NMR spectra (101 MHz, CDCl_3_) of 51**

**^1^H NMR spectra (400 MHz, CDCl_3_)**

**^13^C NMR spectra (101 MHz, CDCl_3_)**
